# Genetic evidence from Indian red jungle fowl corroborates multiple domestication of modern day chicken

**DOI:** 10.1186/1471-2148-8-174

**Published:** 2008-06-10

**Authors:** Sriramana Kanginakudru, Muralidhar Metta, RD Jakati, J Nagaraju

**Affiliations:** 1 Laboratory of Molecular Genetics, Centre for DNA Fingerprinting and Diagnostics, ECIL-Road, Nacaharam, Hyderabad, 500076, India; 2 Van Bhavan, Plot No 6-18, Sector 6, Panchakula, Haryana, India; 3 Department of Neurobiology, University of Massachusetts Medical School, Worcester, MA 01605, USA

## Abstract

**Background:**

Domestication of chicken is believed to have occurred in Southeast Asia, especially in Indus valley. However, non-inclusion of Indian red jungle fowl (RJF), *Gallus gallus murghi *in previous studies has left a big gap in understanding the relationship of this major group of birds. In the present study, we addressed this issue by analyzing 76 Indian birds that included 56 *G. g. murghi *(RJF), 16 *G. g. domesticus *(domestic chicken) and 4 *G. sonneratii *(Grey JF) using both microsatellite markers and mitochondrial D-loop sequences. We also compared the D-loop sequences of Indian birds with those of 779 birds obtained from GenBank.

**Results:**

Microsatellite marker analyses of Indian birds indicated an average F_ST _of 0.126 within *G. g. murghi*, and 0.154 within *G. g. domesticus *while it was more than 0.2 between the two groups. The microsatellite-based phylogenetic trees showed a clear separation of *G. g. domesticus *from *G. g. murghi*, and *G. sonneratii*. Mitochondrial DNA based mismatch distribution analyses showed a lower Harpending's raggedness index in both *G. g. murghi *(0.001515) and in Indian *G. g. domesticus *(0.0149) birds indicating population expansion. When meta analysis of global populations of 855 birds was carried out using median joining haplotype network, 43 Indian birds of *G. g. domesticus *(19 haplotypes) were distributed throughout the network sharing haplotypes with the RJFs of different origins.

**Conclusion:**

Our results suggest that the domestication of chicken has occurred independently in different locations of Asia including India. We found evidence for domestication of Indian birds from *G. g. spadiceus *and *G. g. gallus *as well as from *G. g. murghi*, corroborating multiple domestication of Indian and other domestic chicken. In contrast to the commonly held view that RJF and domestic birds hybridize in nature, the present study shows that *G. g. murghi *is relatively pure. Further, the study also suggested that the chicken populations have undergone population expansion, especially in the Indus valley.

## Background

Archeological findings have indicated that the 'mother of all poultry' is the Southeast (SE) Asian Red jungle fowl (RJF) (*Gallus gallus*). Since domestication of chicken has been observed at the Indus valley as early as 3,200 BC, it is believed to be the epicenter of chicken domestication [1]. However, later day excavations in Peiligan Neolithic sites of China have raised questions about the exclusive domestication at Indus valley, suggesting alternate and possibly earlier domestication centers [2]. It is proposed that *G. gallus*, the wild RJF found in the forests of SE Asia and India, spread to other parts of the world when people domesticated the chicken, resulting in many chicken breeds [3,4]. Subsequent to domestication, the extensive breeding programmes have resulted in sixty or so breeds of chicken representing four distinct lineages: egg-type, game, meat-type and bantam [5]. While some authors suggest monophyletic origin of domestic chicken [6,7], others provide evidence for multiple and independent domestication events [8]. Such inconsistent observations are attributable to the fact that the initial studies were done with relatively small set of samples. In all these reported studies the native RJFs of Indian sub-continent, *G. g. murghi *were not represented in the analyses due to lack of sequence or molecular marker information on this group of birds.

Taxonomically, genus *Gallus *is composed of four species, *G. gallus *(RJF), *G. lafayettei *(Lafayette's JF), *G. varius *(Green JF) and *G. sonneratii *(Grey JF – GJF). Presently there are 5 sub-species of RJF, *G. g. gallus *(SE Asian RJF), and *G. g. spadiceus, G. g. bankiva, G. g. murghi *(Indian RJF) and *G. g. jabouillei *[9]. These classifications are mainly based on phenotypic traits and geographic distribution of the populations. In literature, wild and domesticated birds are often referred to as 'fowls' and 'chicken', respectively. The domestic chicken is considered either as a sub-species of RJF (*G. g. domesticus*) or as a separate species, *G. domesticus*. However, tight clustering of the different sub-species discounts this existing taxonomical hierarchy [6] rendering sub-species status within RJF redundant.

Besides the taxonomical intricacies, the researchers are also concerned about the genetic integrity and conservation status of the RJF in the wild and those held in avicultural collections. It is suspected that the domestic chicken is hybridizing with the wild RJF resulting in erosion of genetic purity of the wild birds [4,10,11]. Most of these earlier studies are based on either phenotypic characters or DNA analyses confined to small samples. Phylogenetic analyses of mitochondrial D-loop sequence and nuclear genes have indicated possible hybridization between GJF-RJF/domestic birds [11]. In the light of these reports it is important to assess the genetic uniqueness of Indian RJFs not only from conservation point of view, but also for using them in population studies.

It is well known that the patterns of genetic differences can reveal the demographic history of the population under study. Since changes in population size leave characteristic molecular signatures, by measuring such changes one can reconstruct the population history. Mismatch distributions, also known as 'pairwise differences' give information on genetic differences between pairs of subjects and can be used to understand the population history [12]. Mitochondrial DNA (mtDNA) is inherited maternally [13] and is often employed in population genetic analyses due to its high copy number, haploid nature and absence of/rare recombination events [14]. A recent study involving the analysis of chicken mitochondrial DNA sequence from pre-historical samples pointed pre-columbian Polynesian origin of American breeds [15], thus suggesting the importance of mitochondrial D-loop sequence in determining the history of chicken domestication. Microsatellite markers, on the other hand, are nuclear markers and are used extensively in population genetic analyses because they are highly polymorphic, ubiquitously distributed throughout the genome, are having high mutation rates, co-dominant in nature, selectively neutral and are amenable to PCR-based high through-put analysis [16]. Hilel et al. [17] characterized 52 chicken breeds using 22 microsatellite markers and concluded that the origin of domestic chicken to be from RJFs, as supported by mt DNA analyses [8].

In all the earlier studies these two marker systems have been used independently to study chicken populations [7,17-19]. In the present study we have combined the results emanating from these two informative marker systems to address the questions relating to (i) evolutionary status of Indian RJF and chicken and (ii) extent of gene flow between Indian RJF and chicken, RJF-GJF in comparison to the world population.

## Results

### Genetic identity of Indian fowls

In the present study, phylogenetic and demographic profiling analyses were carried out using 76 Indian birds that belong to seven populations. We employed 11 microsatellite markers and also sequenced 650 bp of hyper variable region (D-loop) of mitochondrial genomes of two species of fowls namely *G. sonneratii *(n = 4) and *G. gallus*, which includes two subspecies *G. g. murghi*, (n = 56) and *G. g. domesticus *(n = 16).

Totally there were 197 alleles from 11 microsatellite loci, of which, 106 were from *G. g. murghi*, and 59 were from *G. g. domesticus *chicken. The locus MCW5 gave the maximum number of alleles in *G. g. murghi *and in *G. sonneratii*, while VITIIG2 locus revealed the maximum number of alleles in *G. g. domesticus *(Table 1). The total number of private alleles was maximum in *G. g. murghi *followed by *G. g. domesticus *and *G. sonneratii*, respectively. Population-wise mean number of alleles per locus ranged from 2.91 (*G. sonneratii*) to 6.09 (Birshi Kargah population of *G. g. murghi*, B-RJF), with a mean heterozygosity ranging from 0.481 (K-RJF) to 0.600 (B-RJF).

**Table 1:** Microsatellite loci and PCR conditions used in the present study, and population-wise number of alleles obtained for Indian chicken. Original references are listed in the table.

Locus	Repeat motif	Allele Size range	**Tm **°C	MgCl_2_	Total no. of alleles*			Reference
					*G. sonneratii*	*G. g. murghi*	*G. g. domesticus*	
ADL210	(AC)_15_	116–130	46	1.5 mM	1	2	5	[41]
CALB1	(T)_25_	76–106	55	2.5 mM	2	3	3	[42]
HSF3A	(GAG)_10_	230–259	55	1 mM	2	12	8	[42]
HUJ1	(GT)_23_	150–180	55	1.5 mM	4	11	7	[43]
MCW1	(GT)_9_	157–171	62	1.5 mM	1	6	5	[42]
MCW305	(GT)_13_	250–275	55	1.5 mM	3	3	7	[43]
MCW306	(AT)_11_	142–176	55	1.5 mM	4	9	6	[43]
MCW4	(TG)_8_	146–188	67	2.5 mM	2	15	3	[18]
MCW5	(TG)_6_AA(TG)_6_(A)_15 _(GA)_2_(GAA)_21_	184–263	62	1.5 mM	4	18	9	[18]
MYCN	(TG)_17_	174–210	69	2 mM	4	12	5	[43]
VITIIG2	(TTTG)_6_	143–179	51	3 mM	5	15	1	[41]

**Total**					**32**	**106**	**59**	

*No. of alleles obtained in the present study.

When the 76 birds were subdivided into seven groups (see methods section for the grouping of birds) and analyzed for Hardy-Weinberg equilibrium (HWE), of the 77 combinations (7 groups × 11 loci) 32 deviated from HWE, with 22 deviations occurring in *G. g. murghi *alone and 8 in *G. g. domesticus *group (See Additional file 1). We speculate that *G. g. murghi *is probably not in HWE because of inbreeding within the fragmented populations. The low F_ST _values among *G. g. murghi *as compared to *G. g. domesticus *birds also suggested possible inbreeding within *G. g. murghi *populations. The average F_ST _was 0.126 within *G. g. murghi*, while it was 0.154 within *G. g. domesticus *(Table 2). The AMOVA estimation based on 99 permutations using GenAlEx showed a significant (*P *= 0.01) within population variation (66% in 7G and 69% in 2G population). Upon grouping the 72 Indian birds as domestic and RJF, the microsatellite markers showed a significant variation 'among the groups' (i.e. domestic-RJF, 27%, *P *= 0.01) than 'within the group' (17%, *P *= 0.01).

**Table 2:** Population pairwise F_ST _values of microsatellite (below diagonal) and mitochondrial D-loop sequence (above diagonal) for 76 Indian chicken classified into 7 groups.

	M-RJF	B-RJF	K-RJF	C	J	M	GJF
**M-RJF**		0.098	0.095	0.277	0.418	0.329	0.777
**B-RJF**	0.123		0.128	0.342	0.41	0.339	0.798
**K-RJF**	0.140	0.114		0.505	0.627	0.502	0.844
**C**	0.303	0.259	0.280		0.332	0.277	0.787
**J**	0.311	0.246	0.306	0.164		0.306	0.835
**M**	0.299	0.251	0.307	0.141	0.157		0.824
**GJF**	0.210	0.190	0.218	0.316	0.327	0.302	

Note: RJF – *G. g. murghi *populations. M – Morni Hill, B – Birshi Kargah, K – Kalesar. C, J and M represent *G. g. domesticus *from Chicken, Jodhpur and Mirpur Bakshiwala populations. GJF – *G. sonneratii *(India).

Population-wise Nei's genetic distance calculated using GenAlEx program showed a higher average distance between *G. sonneratii *and *G. g. domesticus *(2.099) than between *G. sonneratii *– *G. g. murghi *(0.758) and *G. g. murghi *– *G. g. domesticus *(1.695) combinations (Additional file 1). Intra-population average distances were lower for both domestic and RJF groups than across the population distances, which is consistent with the observation that among population variation was more than within population variation. The results suggest very rare genetic exchange between the RJF and domestic chicken populations, at least in recent history.

**Figure 1:**
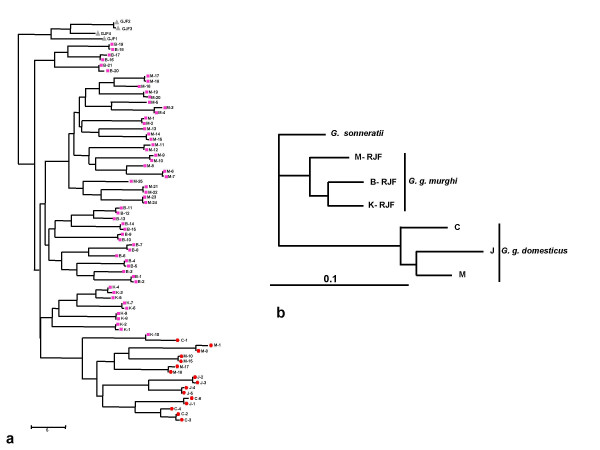
**a) A Maximum likelihood phylogenetic tree constructed using microsatellite data of Indian birds.** Contml program of Phylip package was used to construct a ML tree. The birds segregate according to the groups as domestic (red hexagonal), RJF (pink square) clades with GJF as an outgroup (grey triangle). Scale indicates the allele frequency. b) A neighbor-joining phylogenetic tree constructed based on genetic distance using microsatellite data of Indian birds. Genetic distance was calculated with GenAlEx program and the distance matrix was used to construct NJ tree in MEGA with K2P parameter. The distinct clades of *G. g. murghi *and *G. g. domesticus *is evident with *G. sonneratii *forming the outgroup. Scale indicates the genetic difference.

A Maximum Likelihood (ML) tree obtained from the microsatellite data showed a clear separation of *G. g. domesticus *from the *G. g. murghi*, with *G. sonneratii *as an outgroup (Fig. 1a) suggesting the genetic distinctness of *G. g. murghi*. However, rare instances of hybridization between Indian RJF and domestic birds cannot be excluded in nature as seen in one case (K10-C1, Fig. 1a).

We also constructed a genetic distance based neighbor-joining (NJ) tree to obtain the genetic relationship among Indian birds. The result clearly points to the fact that hybridization between Indian chicken and Indian RJF *G. g. murghi *in the wild is extremely rare (Fig. 1b).

**Figure 2:**
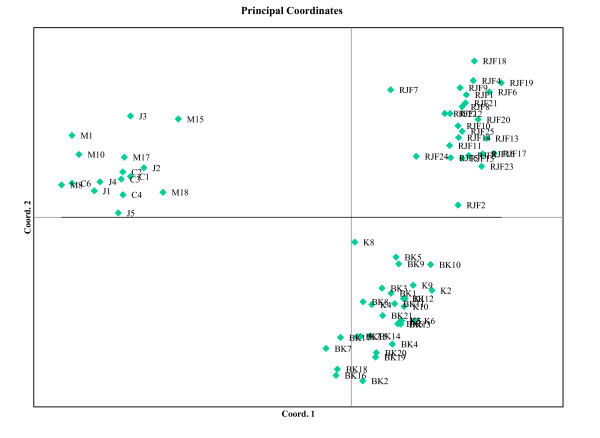
**Principal component analysis based on genetic distances of Indian chicken.** The clear clustering of all domestic chicken to a single quadrate (upper left) indicates absence of hybridization between domestic chicken and RJFs.

To understand the clustering pattern of birds, we also carried out genetic distance based principal component analysis (PCA) (Fig. 2) of domestic and RJF birds, using GenAlEx. The results showed clear segregation of all domestic birds into a single quadrate of the PCA (left upper quadrate in Fig. 2), which did not include any RJF. However, the population of RJFs did not segregate according to the location/population, suggesting the possibility of inbreeding within the RJF populations. This result further supports the absence of hybridization between RJF and domestic birds in India, at least in recent times.

We also sequenced and analyzed 650 bp of the D-loop region of 76 Indian birds to derive the matrilineal population history by using coalescent-based models.

The pairwise F_ST _values calculated using Arlequin were very low within *G. g. murghi *when compared to *G. g. domesticus *(Table 2). The average F_ST _values were more for *G. sonneratii *– *G. g. domesticus *combination than for *G. sonneratii*-*G. g. murghi *or *G. g. murghi *– *G. g. domesticus *combinations. Contrary to microsatellite based analysis, mitochondrial analysis showed a lower mean pairwise F_ST _value for *G. sonneratii *– *G. g. domesticus *combination. These results again point out that there is hardly any genetic exchange across the three categories of birds, namely GJF, RJF and domestic birds.

**Figure 3:**
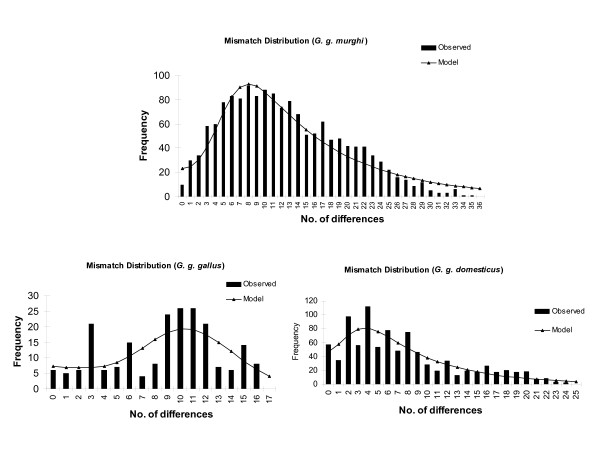
**Mismatch distribution of *G. g. murghi, G. g. domesticus *(India) and *G. g. gallus*.** Observed and model frequencies are indicated. A bell shaped curve characteristic of expanding population is seen for *G. g. murghi*.

To understand the demographic history of the two populations, we obtained pairwise mismatch distribution estimates. In an expanding population, mismatch distribution is expected to be a bell shaped smooth curve, whereas population at constant growth shows raggedness. In the present study, observed mismatch distribution was bell-shaped for 56 Indian birds of *G. g. murghi *(Fig. 3). However, appearance of multiple peaks suggested a population subdivision, which was more prominent in *G. g. domesticus *than in *G. g. murghi*. This also suggests homogenous population of *G. g. murghi*, again supporting our notion that inbreeding is more common in RJF than in domestic birds. Harpending's raggedness index was lower in *G. g. murghi *(0.001515) than in *G. g. domesticus *(0.0149), but was significant (P < 0.05) in both the populations reiterating population expansion.

### Phylogeny and median-joining network profiles of Indian birds in comparison to global populations

In the present study we also compared the D-loop sequence of 76 birds with the corresponding sequences (approximately 400–440 bp) of 779 birds from NCBI GenBank to get insight into phylodemographic status of *G. g. murghi *and *G. g. domesticus *(India). Of the 855 sequences, 117 were from RJFs, 714 were from domestic chicken and the rest belonged to other species of *Gallus*. These 855 birds were classified into 13 groups based on geographical location and species/sub-species status as shown in Table 3. We included Japanese quail, *Coturnix japonica *as an outgroup whenever necessary.

**Table 3:** Standard diversity indices and mismatch distribution analyses obtained from d-loop sequence of 855 birds belonging to 13 populations. *Coturnix japonica *that was used as an outgroup is not included.

Group→	*G. gallus domesticus *(Domestic chicken)					*G. gallus *(RJF)				Other species of *Gallus*			
			
	India	Indonesia	Japan	Misc.*	China	*murghi*	*gallus*	*spadiceus*	*Bankiva*	*G. jabouillei*	*G. Varius*	*G. lafayettei*	*G. sonneratii*
Sample size	43	12	104	4	551	56	21	41	6	3	4	3	6
Haplotypes	23	6	26	-	82	42	15	12	6	-	-	-	5
Haplotype diversity (Hd)	0.932	0.836	0.925	-	0.934	0.948	0.971	0.789	1.000	-	-	-	0.933
Sum of square freqs.	0.023	0.083	0.010	0	0.002	0.018	0.048	0.024	0.167	0	0.250	0.333	0.167
Number of observed transitions	30	16	30	17	49	45	31	23	8	14	7	4	33
Number of observed transversions	20	0	3	0	9	47	3	4	0	1	3	1	2
Number of substitutions	50	16	33	17	58	92	34	27	8	15	10	5	35
Number of polymorphic sites	55	16	37	17	55	113	36	29	10	15	75	5	39
Nucleotide diversity Standard deviation (±)	0.018 0.009	0.015 0.008	0.021 0.011	0.024 0.016	0.018 0.009	0.029 0.015	0.022 0.011	0.023 0.011	0.012 0.008	0.025 0.019	0.082 0.054	0.007 0.006	0.034 0.020
Mismatch observed mean	7.409	6.000	8.670	9.500	7.354	12.436	8.800	9.229	5.000	10.000	38.000	3.333	16.000
Harpending's Raggedness index	0.021	0.236	0.012	0.167	0.009	0.001	0.029	0.136			0.750	1.000	0.289
*P *(Sim. Rag. > = Obs. Rag.)	0.310	0.000	0.230	0.960	0.310	1.000	0.300	0.000			0.120	0.860	0.160
Tajima's D	-1.406	0.571	0.835	0.250	-0.148	-1.923	-0.352	1.518	0.947	0	-0.222	0	-0.357
P(D random < D obs)	0.075	-0.293	-0.204	-0.321	0.470	0.015	0.383	-0.073	-0.204	0	0.540	0	0.420
Fs	-24.995	-7.215	-24.627	0.273	NC	-24.401	-14.732	-24.738	-2.160	1.139	1.798	-0.077	-0.284
Prob(sim_Fs < = obs_Fs)	0	0.001	0	0.319	-	0	0	0	0.049	0.46	0.496	0.239	0.233

* Misc. represents birds from Iran, UK and Vietnam. NC – not calculated.

**Figure 4:**
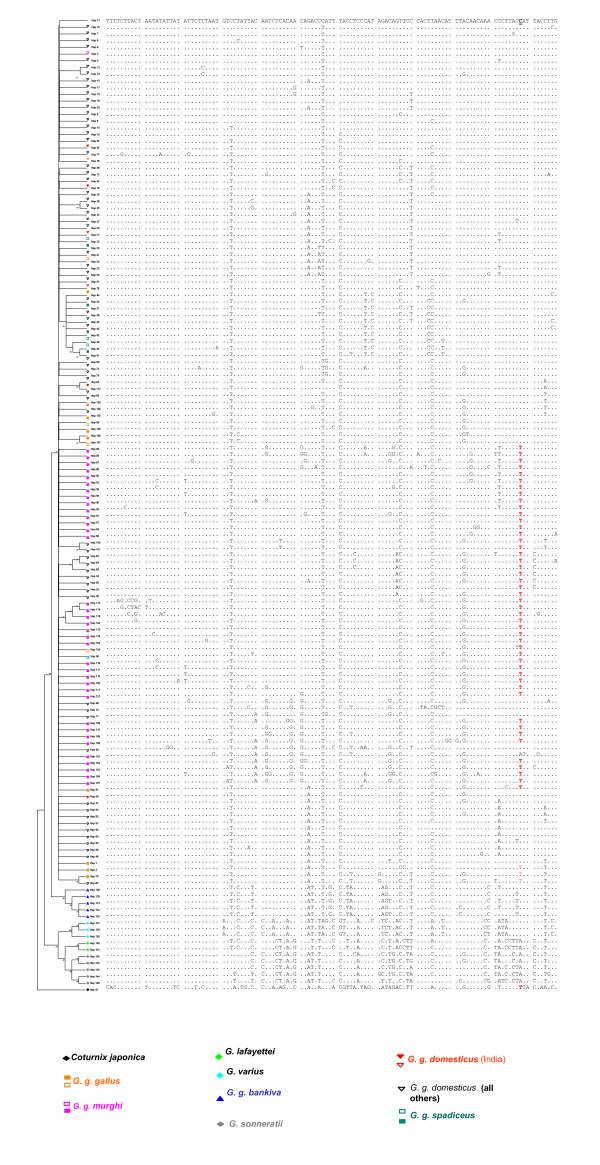
**A NJ haplotype tree obtained by 50% consensus rule using 117 variable sites within the sequenced portion of the D-loop, with their corresponding haplotypes generated from the 146 haplotypes of 855 birds.** Different haplotypes are color-coded based on the group they belong to (See methods section for grouping details) as indicated. Filled structures indicate haplotypes consisting of all birds of the same group (e.g. Indian RJF-pink colored), while open structures indicate the presence of at least one bird of a specified/color-coded group that also contains birds belonging to other groups (e.g. Indian RJF containing domestic birds). Different sub-species of *G. gallus *form only two clusters, one of *G. g. bankiva *and others containing all other sub-species. The cluster of *G. gallus *sub-species, however also contained another species of *Gallus*, namely *G. lafayettei*. Identical sequences are shown as dots in comparison with H_11. A specific mutation found in 96% of *G. g. murghi *is shown in bold and red color and the corresponding nucleotide is underlined in H_11. This position corresponds to the nucleotide number 360 (where there is 'C') in the complete mitochondrial genome sequence of *G. gallus *(Acc. No. NC_001323). The color-coding used is represented below the figure.

All the 855 sequences resulted in 117 segregating sites and 146 haplotypes with a haplotype diversity of 0.940 and an average nucleotide diversity of 0.020. The NJ tree generated from the haplotype data revealed two distinct *G. gallus *clusters, one consisting of the *G. g. bankiva *group, and the remaining cluster containing all the other groups namely, *G. g. spadiceus, G. g. gallus, G. g. murghi, G. g. jabouillei *and *G. g. domesticus*. Irrespective of the geographic distribution and sub-species status, more than 95% of the birds clustered within this single largest group (Fig. 4 – see discussion). This result is consistent with the neighbor-joining tree of all 855 birds (Additional file 2), where we observed a single clade for all domestic and RJF birds. However, in contrast to Fumihito et al.'s work [5], we observed a clear segregation of a clade containing *G. varius, G. lafayettei *and *G. sonneratii *from all RJF/domestic bird combination, which reassures us about the absence of the hybridization. Such a topology was also evident from the haplotype-based tree (Fig. 4)

**Figure 5:**
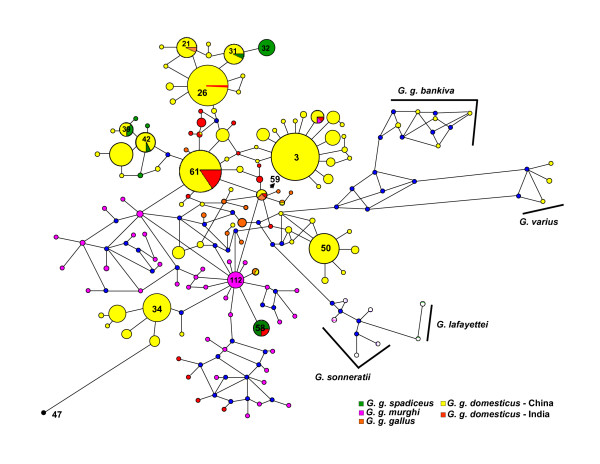
**Median-joining haplotype network of 855 birds belonging to 146 haplotypes.** Various haplotypes of chicken species/sub-species are represented in different colored circles. The size of the circles is proportional to the haplotype frequency. Some of the important haplotype numbers are indicated. The median vectors that represent hypothetical intermediates or un-sampled haplotypes, are shown in blue circles. The data indicate formation of a star-like phylogeny of *G. g. murghi *around H_112. Black circle (H_47) indicates *C. japonica*. Other *Gallus *species are indicated by their names.

To obtain further insight into the haplotype relationships, we constructed a median-joining network using 146 haplotypes. In the haplotype network, most of the *G. g. gallus *formed individual nodes (e.g. H_1, H_2, H_78, H_79) and clustered independently, while many *G. g. murghi *haplotypes (H_86 to H_99 and H_102 to H_123) grouped around a major cluster (H_112) having 16 birds, giving rise to a star like phylogeny (Fig. 5). The largest haplotype H_3 (with 127 birds) confined mostly to Chinese *G. g. domesticus *birds without any representation of jungle fowls. Thirty nine birds of *G. g. spadiceus *belonging to 9 haplotypes did not have any representation from *G. g. murghi *birds. The second largest haplotype (H_61 with 102 birds) shared only a single individual of *G. g. gallus *and many *G. g. domesticus *from China, India, Japan and Indonesia. Forty-three *G. g. domesticus *of India (19 haplotypes) were distributed throughout the network. These haplotypes were connected with *G. g. gallus, G. g. murghi *and *G. g. spadiceus*. The haplotype H_63 contained *G. g. domesticus *(India) birds reported in the present study as well as from an earlier study [8]. Interestingly, majority of the Indian *G. g. domesticus *haplotypes shared the birds from the three major *G. gallus *groups belonging to *G. g. spadiceus *(H_58, H_61), *G. g. gallus *(H_59, H_61), and also *G. g. murghi *(H_6). It is very likely that *G. g. spadiceus *has also contributed to the domestic chicken. Apart from Indian RJF, *G. g. murghi *haplotype H_6 also contained 5 Chinese, 1 Iranian and 1 Japanese domestic chicken. The distribution of Indian domestic chicken into different haplotypes could imply multiple origins of Indian domestic chicken breeds.

The sequenced regions of D-loop were well conserved in most of the birds. The 62 bp insertion element found in *G. sonneratii *was the only indel of considerable size present in the whole sequence. The salient mutation observed in *G. g. murghi *was the presence of 'T' in most of the birds (54 out of 56 i.e. 96.4%) (Fig. 4) that was present in only 33.3% of *G. g. gallus*, 24.4% of *G. g. spadiceus *and 13.1% of all the *G. g. domesticus *birds. Only three Indian *G. g. domesticus *(6.9%) had 'T' at this position. This position corresponds to the nucleotide number 360 (where there is 'C') in the complete mitochondrial genome sequence of *G. gallus *(Acc. No. NC_001323) available in GenBank. Across the portion of the sequenced mitochondrial genome we observed that transition to transversion ratio was lower in case of *G. g. murghi *(45/47 = 0.99) than in *G. g. gallus *(31/2 = 15.5) or *G. g. spadiceus *(23/4 = 5.75 – Table 3).

Mismatch distribution analyses carried out under sudden expansion model showed that mean pairwise differences were highest in *G. g. murghi *among *G. gallus *sub-species. However, another species of *Gallus, G. sonneratii *had the highest mean pairwise differences (Table 3). Both Tajima's D and Fu's Fs were significantly (*P *< 0.05) negative in *G. g. murghi*, suggesting the departure from neutrality (Table 3). Tajima's D was significant only in *G. g. spadiceus *and *G. g. murghi*. Fu's F_S_, a better indicator for estimating the departures from neutral theory, showed a significant negative value (*P *of simF_S _< = obsF_S _is < 0.01) for *G. g. murghi *and *G. g. domesticus *(Indonesia). Nucleotide diversity was also high (0.030) in case of *G. g. murghi *(except for *G. sonneratii*) amongst all the groups studied (Table 3).

The F_ST _values showed a very high differentiation between the out group *C. japonica *and the other 13 populations, with a value above 0.9. Most of the pairwise F_ST _values were significant with a *P *value < 0.05. F_ST _value [20] based NJ tree showed the divergence of *G. g. murghi *from *G. g. gallus, G. g. spadiceus *and *G. g. domesticus *(Additional file 3). AMOVA calculations showed that the majority of variation found within *G. gallus *subspecies was between the domestic and jungle fowl populations (76%), while 'among the group' variation was 7% with an overall F_ST _value of 0.234 (*P *= 0).

## Discussion

In the present study we attempted to understand the contribution of Indian red jungle fowl, *G. g. murghi *to the domestication event. As of now, no sequence information is available from this group of birds which, in all likelihood, was contributor of one of the earliest known chicken domestication event, i.e. in Mohanjo-Daro. We studied Indian birds belonging to two species of *Gallus *and compared them with the worldwide bird populations. Since microsatellite markers and the D-loop sequence of mitochondrial DNA have a high mutation rates, they provide information about recent evolutionary history as compared to slow mutating genes that provide data about ancient history [21]. To reconstruct the recent past, we used both these marker systems and also addressed the issue of genetic purity of wild birds.

From the mtDNA analysis, we observed that a group of *G. g. domesticus *birds had the *G. g. murghi *haplotype (H_6), while a few others shared haplotypes with *G. g. gallus *(H_21, H_59, H_61 etc.) and with *G. g. spadiceus *(H_39, H_42, H_58 etc.). This is also true for Indian chicken that have originated by independent domestication from *G. g. murghi *as well as possibly from other *G. g*. subspecies. Interestingly, sharing of different haplotypes by Indian domestic chicken clarify that the present day Asiatic chicken might have originated from different progenitors by multiple domestication events and such multi-origin breeds could still be observed in a single geographical location. This is consistent with the observation of Oka et al. [22], who showed that the present day native Japanese chicken are having multiple origin.

**Figure 6:**
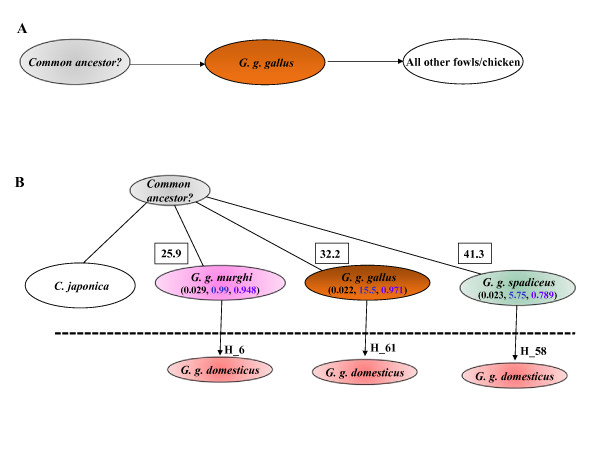
**Comparison of (A) single domestication against (B) multiple domestication hypothesis.** The present study supports the hypothesis B for origin of domestic chicken. The numbers in parenthesis indicate the nucleotide diversity (p-black), transition-to-transversion ratio (Ts/Tv-blue) and haplotype diversity (Hd-violet), respectively. The number inside the square indicates average number of mutational events of each group from the outgroup, *C. japonica*. The sharing of the haplotypes (as exemplified by H_number) indicates the multiple origin of the domestic chicken from different jungle fowls. Low mutational distance, low Ts/Tv ratio and high nucleotide diversity indicate the ancient nature of Indian RJF, *G. g. murghi*. The dashed line separates domestic chicken (below the line) from other birds.

A model explaining the origin of Indian domestic bird by multiple domestications is depicted schematically in Fig. 6. Sharing of haplotypes, as indicated in this model, suggests multiple origins to Indian domestic chicken. Independent domestications have also been reported for cattle [23], pig [24] suggesting that such events are not rare. However, all the birds except *G. g. bankiva *form a single cluster suggesting a common ancestor long back in history for these birds including jungle fowls and domestic birds. The separation of *G. g. bankiva *from the main cluster of birds indicates the possibility of a speciation event.

The multilocus microsatellite data as well as the D-loop sequence of Indian chicken showed departure from neutrality as indicated by significant negative value of Tajima's D and Fu's Fs for *G. g. murghi *suggesting the possible population expansion of Indian birds. These results are also consistent with mismatch distribution analyses and significant value for Harpending's raggedness index (Table 3). Taken together with the genetic diversity analyses, we surmise that *G. g. murghi *and *G. g. domesticus *(India) must have undergone population expansion. It is believed that population expansion follows a domestication event. This fact holds true even in case of chicken, where we did observe population expansion, as did previous studies.

Our analyses revealed that the sampled Indian birds are relatively pure with very rare hybridization between *G. g. murghi *and *G. g. domesticus *(India). Nishibori et al. [11] suggested the hybridization of RJF with domestic and grey jungle fowl. In the present study of Indian birds, we did not come across noticeable hybridization at least in the recent past, as indicated by very low F_ST _values for mtDNA (Table 2) and microsatellite markers and also a clear separation of RJF clades from domestic chicken in microsatellite based phylogeny. All these results indicate the genetic integrity of the *G. g. murghi*.

In the present study, we observed predominant occurrence of a characteristic 'T' nucleotide in 96.4% of *G. g. murghi *birds that is absent in most of the Indian domestic chicken further supporting the occurrence of negligible hybridization between *G. g. murghi *and *G. g. domesticus *(India). All the sampled *G. sonneratii *had 'A' nucleotide in this position. If the frequent hybridization is occurring then it is expected that at least a few of *G. g. murghi *to have 'A' at this position. Since we found only one RJF with 'A' at this site, and also due to clear separation of the clades, it is unlikely that *G. g. murghi *and *G. sonneratii *hybridize in the wild contrary to the observations made by Nishibori et al. [11] who suggested the possible hybridization between the RJF and GJF. Such contrasting observations may be due to the limited number of samples (3 RJF and 3 GF) used by Nisibori et al. [11]. Taken together with the Fumihito et. al.'s [6,7] and Liu et al.'s [8] observations our results prompted us to question the sub-species status for *G. g. gallus, G. g. spadiceus, G. g. murghi *and *G. g. domesticus*. In the light of these findings, we recommend that *G. gallus *should be classified as *G. g. gallus *that should include all RJFs and *G. g. domesticus *birds. At the same time, after confirmation of reproductive isolation, *G. g. bankiva *could be placed into a separate species, *Gallus bankiva*.

## Conclusion

For the first time by analyzing hitherto unreported samples of *G. g. murghi *and also including the *G. g. domesticus *(India), we confirm that the domestication of chicken has occurred independently from *G. g. murghi*. We also provide evidence that there is little genetic exchange between *G. g. murghi *and *G. g. domesticus *(India) and minimal hybridization between *G. sonneratii *and *G. g. murghi*. Comparison of Indian RJF and domestic bird to that of world population also supports the previous studies of obsoleteness of the sub-species status given to RJFs and domestic chicken.

## Methods

### Samples, DNA isolation

**Figure 7:**
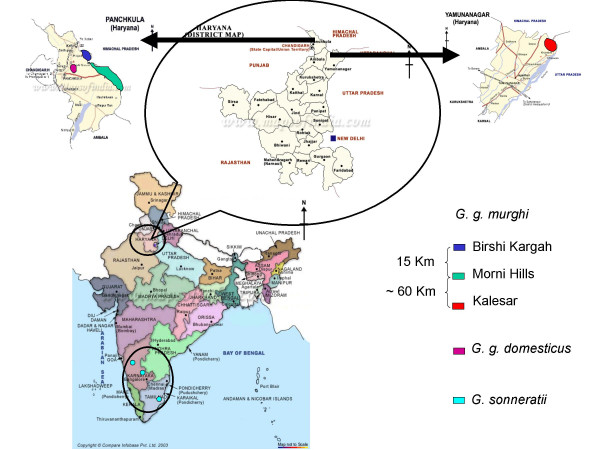
**Map of India showing the sampling locations.** RJFs were collected from two districts, while domestic birds were from the same district of Haryana state in north-west India. Grey jungle fowls were from south India. Distances between the villages are shown in kilometers. The places from where the samples were collected are shown in colored circles. *G. sonneratii *was collected from the south Indian states of Karnataka and Tamilnadu.

Blood samples were collected from 3 geographically isolated populations of *G. g. murghi *i.e. Morni Hills ('M-RJF', n = 25), Kalesar Forest ('K-RJF', n = 10) and Birshi Kargah Forest ('B-RJF', n = 21) of Haryana State of India (Fig. 7), 3 populations of *G. g. domesticus *birds i.e. Chicken ('C', n = 5), Jodhpur ('J', n = 5) and Mirpur Bakshiwala ('M', n = 6) villages of Haryana. Grey Jungle fowl (*G. sonneratii*) blood samples (n = 4) were collected from different Zoological Parks of South India and were considered as a single population. Except for the *G. g. domesticus *birds, which were collected from specific villages, all the other birds were captive bred from the founder-lines that were obtained from the specified village. The 76 birds were considered as belonging to 7 populations (3 RJF, 3 domestic and a single population of GJF).

About 0.5 ml of blood was collected from the wing vein of live birds into vials containing 5 mM EDTA and genomic DNA was isolated as per standard protocols [25].

### PCR amplification of microsatellite loci

The microsatellite loci used in the study, the primer details and PCR conditions are available in Table 1. The PCR products were separated on 3.5% Metaphor agarose gel along with pUC/MspI digest (MBI Fermentas) ladder. For GeneScan analysis, the PCR products generated using fluorescent dUTPs were dissolved in 2 μl of formamide gel loading buffer with 0.3 μl of ROX-500™ GeneScan ruler (Perkin Elmer) and separated on a 5% polyacrylamide-7 M Urea gel.

### PCR amplification and Sequencing of D-loop of mitochondrial DNA

The D-loop hypervariable region was PCR amplified using primers described elsewhere [7]. The sequencing was carried out on both the strands. We also used an internal primer (5'GTGGAATATAGGTTAATGCC 3') to obtain the sequence information from 5' region without any ambiguity. 50 ng PCR product was used in a sequencing reaction that contained 8 μl of Ready reaction mix (BDT v 3.0, Applied Biosystems, Foster City, CA) and 5 picomoles of primer. The sequencing was carried out in ABI Prism 3100 Genetic Analyzer (Applied Biosystems).

### Data analysis

#### Microsatellite Data

The individuals were genotyped based on allele size data. Allele frequency and heterozygosity were calculated using GenAlEx [26]. *F*-statistics were used as a measure of diversity within and between populations and were estimated using GenAlEx. For PCA, genetic distance was calculated from the allele data and the genetic distance was plotted as PCA using GenAlEx. Population history parameters were calculated using Arlequin [27]. The microsatellite allele frequency data from different populations was bootstrapped using seqboot of Phylip and the output file was used for construction of maximum likelihood (ML) tree using Phylip 'contml' program. For distance based NJ tree, the genetic distance was calculated using GenAlEx program and the resulting distance matrix was used to construct NJ tree with Kimura-2-parameter (K2P) option in MEGA.

#### Mitochondrial DNA

Mitochondrial D-loop sequences of 779 Jungle Fowls and *G. g. domesticus *birds as well as other species of *Gallus *were obtained from GenBank [28] (available as Additional file 4). The sequences of 855 birds, including the 76 samples sequenced in the present study were aligned using ClustalX program [29], manually edited using GeneDoc [30] and the region conserved in all the birds was used for analysis. With gaps there were a total of 482 bp sequence, with highest number of nucleotides (460–462) coming from the *G. sonneratii *and approximately 400 bp from RJFs. Similar to previous phylogenetic studies, we used *Coturnix japonica *as an outgroup in our study. The phylogenetic trees were constructed using Phylip 3.5 [31] or MEGA [32]. The best fit model was selected using the program MODELTEST [33] as implemented in HyPhy [34]. The alpha value obtained from best-fit model was used for gamma correction in haplotype NJ tree, which was constructed using Tajima and Nei's model in MEGA3. Haplotype data was obtained using DnaSP [35] and was used to construct haplotype network using Network program [36]. Default parameters were used for obtaining the median joining network tree. Population genetic structure was measured using AMOVA as implemented in Arlequin with 1000 permutations. Tajima's D [37], Fu's Fs [38] and other population genetics parameters were also calculated using Arlequin for which the significance was tested after 1000 simulation steps [27]. For such analyses, populations were defined depending on the data being analyzed – e.g. in case of Indian chicken, as 2G – 2 populations *viz. G. g. domesticus *and *G. g. murghi*, 6G-3 sub populations each based on the sampling location in both *G. g. domesticus *(C, J, M) and *G. g. murghi *populations (M-RJF, B-RJF, K-RJF) or 7G (6G and *G. sonneratii*). Such a classification was carried out to study the population genetic structure within the subdivided populations. Whenever necessary, as in case of subdivided populations, realignment of the sequence was carried out using clustalX.

We investigated the demographic profiles of chicken populations based on coalescence theory and analyzed pairwise mismatch distribution to confirm the population expansion [39], using Arlequin. The parameter of demographic expansion τ was estimated with a generalized nonlinear least squares approach and approximate confidence intervals were obtained with 1000 parametric bootstrap replicates. The goodness-of-fit of the observed data to a simulated model of expansion was tested with the sum of squared deviations and Harpending's raggedness index was estimated [40]. For bootstrap phylogenetic NJ tree, the aligned mtDNA sequence was run in MEGA with 1000 replicates with a 50% cutoff option.

## Authors' contributions

RDJ carried out sample collection, MM did the PCR and sequencing and contributed to analyses, SK carried out the computational and phylogenetic analyses and prepared the manuscript, JN conceived the study, participated in its design and coordination and revision of the manuscript.

## Supplementary Material

Additional file 1 **Table S1**. GenAlex file showing the Nei's genetic distance, HWE and allele frequencies. Click here for file

Additional file 2 **Fig. S1**. A bootstraped NJ tree of all 855 birds obtained using MEGA. The value indicates the bootstrap support. The names of individual birds has been modified, hence may not exactly correlate with Table S2. Click here for file

Additional file 3 **Fig. S2**. F_ST _value based NJ tree showing the divergence of *G. g. murghi *from *G. g. gallus, G. g. spadiceus *and *G. g. domesticus*. Clade I contains all RJFs and domestic chicken subgroups. Click here for file

Additional file 4 **Table S2**. Details of 855 birds used in the present study. Click here for file
