# Supplementary figures and images for: Genetic evidence from Indian red jungle fowl corroborates multiple domestication of modern day chicken
Source: BMC Evol Biol. 2008 Jun 10;8:174. doi: 10.1186/1471-2148-8-174 (PMC2474866; doi:10.1186/1471-2148-8-174)

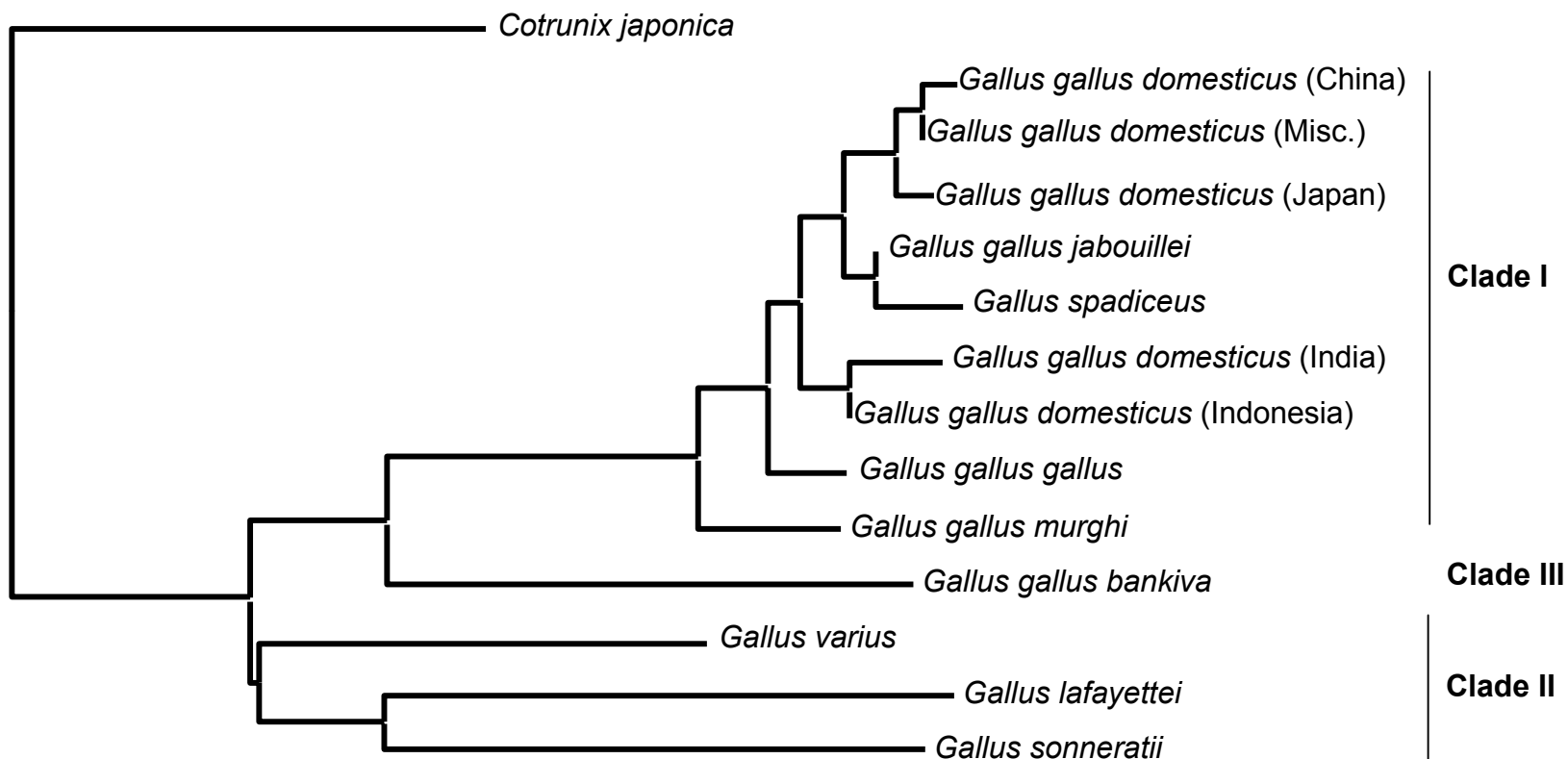

0.1

Supplement: Additional file 3 — Fig. S2. FST value based NJ tree showing the divergence of G. g. murghi from G. g. gallus, G. g. spadiceus and G. g. domesticus. Clade I contains all RJFs and domestic chicken subgroups. [file 1471-2148-8-174-S3.pdf]
