# Supplementary material for: Genetic evidence from Indian red jungle fowl corroborates multiple domestication of modern day chicken
Source: BMC Evol Biol. 2008 Jun 10;8:174. doi: 10.1186/1471-2148-8-174 (PMC2474866; doi:10.1186/1471-2148-8-174)
Supplement: Additional file 4 — Table S2. Details of 855 birds used in the present study. [file 1471-2148-8-174-S4.doc]

| **No.** | **Bird name** | **Accession** | **Class** | **Haplotype** |
| --- | --- | --- | --- | --- |
| 1 | 1 | gi-54399942-AY642134.1| | SE-Dmal1 | Hap_61 |
| 2 | 2 | gi-54399941-AY642133.1| | SE-D2 | Hap_61 |
| 3 | 3 | gi-54399938-AY642130.1| | SE-D3 | Hap_61 |
| 4 | 4 | gi-54399937-AY642129.1| | SE-D4 | Hap_26 |
| 5 | 5 | gi-54399940-AY642132.1| | SE-D5 | Hap_61 |
| 6 | 6 | gi-54399935-AY642127.1| | SE-D6 | Hap_26 |
| 7 | 7 | gi-54399936-AY642128.1| | SE-D7 | Hap_26 |
| 8 | 8 | gi-54399939-AY642131.1| | SE-D8 | Hap_61 |
| 9 | 4.41E+04 | gi-56155378-AY645014.1| | C-409e1 | Hap_65 |
| 10 | 4.24E+05 | gi-56155377-AY645013.1| | C-4235e2 | Hap_61 |
| 11 | 2.72E+07 | gi-56155359-AY644995.1| | C-2715e4 | Hap_61 |
| 12 | 2.74E+08 | gi-56155361-AY644997.1| | C-2739e5 | Hap_61 |
| 13 | 1.81E+11 | gi-56155345-AY644981.1| | C-1812e8 | Hap_67 |
| 14 | 1.93E+12 | gi-56155352-AY644988.1| | C-1927E9 | Hap_61 |
| 15 | 5.19E+12 | gi-56155340-AY644976.1| | C-519e10 | Hap_61 |
| 16 | 3.43E+14 | gi-56155372-AY645008.1| | C-433e11 | Hap_61 |
| 17 | 0515a10 | gi-56155342-AY644978.1| | C-515a10 | Hap_64 |
| 18 | 0517c10 | gi-56155341-AY644977.1| | C-0517c1 | Hap_61 |
| 19 | 0518d10 | gi-56155339-AY644975.1| | C-518d10 | Hap_61 |
| 20 | 0522h10 | gi-56155338-AY644974.1| | C-522h10 | Hap_61 |
| 21 | 10gallus | AB007757 | 10SE-R | Hap_21 |
| 22 | 11gallus | AB007756 | 11SE-R | Hap_79 |
| 23 | 12gallus | AB007753 | 12SE-R | Hap_21 |
| 24 | 13gallus | AB007752 | 13SE-R | Hap_61 |
| 25 | 14gallus | AB007725 | 14SE-R | Hap_124 |
| 26 | 15gallus | D82900 | 15SE-R | Hap_126 |
| 27 | 16gallus | AB007720 | 16SE-R | Hap_1 |
| 28 | 17gallus | D82905 | 17SE-R | Hap_2 |
| 29 | 1804a8 | gi-56155346-AY644982.1| | C-1804a8 | Hap_67 |
| 30 | 1807b8 | gi-56155344-AY644980.1| | CD-1807 | Hap_6 |
| 31 | 1810d8 | gi-56155347-AY644983.1| | C-1810d8 | Hap_61 |
| 32 | 1813f8 | gi-56155343-AY644979.1| | C-1813f8 | Hap_61 |
| 33 | 1815h8 | gi-56155348-AY644984.1| | C-1815h8 | Hap_61 |
| 34 | 18gallus | D82904 | 18SE-R | Hap_1 |
| 35 | 1921a9 | gi-56155351-AY644987.1| | C-1921a9 | Hap_61 |
| 36 | 1923b9 | gi-56155350-AY644986.1| | C-1923b9 | Hap_61 |
| 37 | 1925d9 | gi-56155349-AY644985.1| | C-1925d9 | Hap_61 |
| 38 | 1929c9 | gi-56155353-AY644989.1| | C-1929c9 | Hap_61 |
| 39 | 19Gallus | D82903 | 19SE-R | Hap_1 |
| 40 | 1Baiyiner | AF128320 | 1CD-ba | Hap_21 |
| 41 | 1bankiva | AB009431 | 1bankiva | Hap_132 |
| 42 | 1Gallus | AB009441 | 1SE-R | Hap_127 |
| 43 | 1lafayett | D66893 | 1lafayett | Hap_140 |
| 44 | 1Laihang | AF128329 | 1CD-la | Hap_67 |
| 45 | 1Lingkun | AF128330 | 1CD-L | Hap_21 |
| 46 | 1Nagoya | D82921 | 1J-nag | Hap_11 |
| 47 | 1sonnerat | D82911 | Ind-GJF1 | Hap_142 |
| 48 | 1spadiceu | AB009443 | 1SE-spa | Hap_39 |
| 49 | 1varius | D64163 | 1varius | Hap_137 |
| 50 | 1Wugu | AF128335 | 1CD-wu | Hap_21 |
| 51 | 1Xanju | AF128315 | 1CD-xa | Hap_61 |
| 52 | 1Xiaoshan | AF128344 | 1CD-x | Hap_34 |
| 53 | 20Gallus | D82902 | 20SE-R | Hap_129 |
| 54 | 21gallus | D82901 | 21SE-R | Hap_124 |
| 55 | 2711a4 | gi-56155355-AY644991.1| | C-2711a4 | Hap_61 |
| 56 | 2713c4 | gi-56155354-AY644990.1| | C-2713c4 | Hap_61 |
| 57 | 2714d4 | gi-56155358-AY644994.1| | C-2714d4 | Hap_61 |
| 58 | 2735a5 | gi-56155356-AY644992.1| | C-2735a5 | Hap_61 |
| 59 | 2736b5 | gi-56155357-AY644993.1| | C-2736b5 | Hap_61 |
| 60 | 2737c5 | gi-56155360-AY644996.1| | C-2737c5 | Hap_61 |
| 61 | 2740f5 | gi-56155362-AY644998.1| | C-2740f5 | Hap_61 |
| 62 | 2804b6 | gi-56155363-AY644999.1| | C-2804b6 | Hap_61 |
| 63 | 2806d6 | gi-56155364-AY645000.1| | CD-2806 | Hap_4 |
| 64 | 2Baiyiner | AF128321 | 2CD-ba | Hap_21 |
| 65 | 2bankiva | AB009430 | 2bankiva | Hap_135 |
| 66 | 2Gallus | AB009440 | 2SE-R | Hap_2 |
| 67 | 2Jap1kis | AB007736 | 2J-1kis | Hap_9 |
| 68 | 2Jap2whi | AB007723 | 2J-w | Hap_67 |
| 69 | 2Japbarr | AB007719 | UK-2 | Hap_61 |
| 70 | 2lafayett | D82909 | 2lafayett | Hap_140 |
| 71 | 2Laihang | AF128328 | 2CD-la | Hap_67 |
| 72 | 2Lingkun | AF128331 | 2CD-L | Hap_21 |
| 73 | 2sonnerat | D66892 | Ind-GJF2 | Hap_142 |
| 74 | 2spadiceu | AB009442 | 2SE-spa | Hap_21 |
| 75 | 2varius | D82912 | 2varius | Hap_139 |
| 76 | 2white | D82923 | J-2whi | Hap_67 |
| 77 | 2Wugu | AF128336 | 2CD-wu | Hap_21 |
| 78 | 2Xanju | AF128316 | 2CD-xa | Hap_61 |
| 79 | 2Xiaoshan | AF128343 | 2CD-x | Hap_3 |
| 80 | 3203b7 | gi-56155365-AY645001.1| | C-3203b7 | Hap_61 |
| 81 | 3207f7 | gi-56155366-AY645002.1| | C-3207f7 | Hap_61 |
| 82 | 3313b2 | gi-56155367-AY645003.1| | C-313b2 | Hap_61 |
| 83 | 3317c12 | gi-56155368-AY645004.1| | C-317c12 | Hap_61 |
| 84 | 3319d12 | gi-56155369-AY645005.1| | C-319d12 | Hap_61 |
| 85 | 3425b3 | gi-56155370-AY645006.1| | CD-3425 | Hap_3 |
| 86 | 3427b11 | gi-56155373-AY645009.1| | C-427b11 | Hap_61 |
| 87 | 3436g11 | gi-56155371-AY645007.1| | C-436g11 | Hap_61 |
| 88 | 3AyamPel | D82919 | SE-D3AyP | Hap_59 |
| 89 | 3Baiyiner | AF128322 | 3CD-ba | Hap_21 |
| 90 | 3bankiva | AB007718 | 3bankiva | Hap_136 |
| 91 | 3Gallus | AB009439 | 3SE-R | Hap_1 |
| 92 | 3Jap1sil | AB007735 | 3J-1sil | Hap_9 |
| 93 | 3lafayett | D82910 | 3lafayett | Hap_141 |
| 94 | 3Laihang | AF128327 | 3CD-la | Hap_67 |
| 95 | 3Lingkun | AF128332 | 3CD-L | Hap_21 |
| 96 | 3spadiceu | D82908 | 3SE-spa | Hap_39 |
| 97 | 3varius | D82913 | 3varius | Hap_137 |
| 98 | 3white |  | 3J-w | Hap_66 |
| 99 | 3Wugu | AF128337 | 3CD-wu | Hap_21 |
| 100 | 3Xanju | AF128317 | 3CD-xa | Hap_131 |
| 101 | 3Xiaoshan | AF128342 | 3CD-x | Hap_22 |
| 102 | 4231a2 | gi-56155374-AY645010.1| | C-4231a2 | Hap_61 |
| 103 | 4233c2 | gi-56155375-AY645011.1| | C-4233c2 | Hap_61 |
| 104 | 4234d2 | gi-56155376-AY645012.1| | C-4234d2 | Hap_61 |
| 105 | 4405d1 | gi-56155379-AY645015.1| | C-405d1 | Hap_65 |
| 106 | 4406c1 | gi-56155381-AY645017.1| | C-406c1 | Hap_61 |
| 107 | 4411f1 | gi-56155380-AY645016.1| | C-411f1 | Hap_65 |
| 108 | 4Ayamkok | D82917 | SE-D4kok | Hap_128 |
| 109 | 4Baiyiner | AF128323 | 4CD-ba | Hap_21 |
| 110 | 4bankiva | D82898 | 4bankiva | Hap_134 |
| 111 | 4Gallus | AB009437 | 4SE-R | Hap_125 |
| 112 | 4Jap1nag | AB007722 | J-1nag4 | Hap_11 |
| 113 | 4Laihang | AF128326 | 4CD-la | Hap_67 |
| 114 | 4Lingkun | AF128333 | 4CD-L | Hap_34 |
| 115 | 4spadiceu | D82907 | 4SE-spa | Hap_40 |
| 116 | 4Wugu | AF128338 | 4CD-wu | Hap_21 |
| 117 | 4Xanju | AF128318 | 4CD-xa | Hap_61 |
| 118 | 4Xiaoshan | AF128341 | 4CD-x | Hap_10 |
| 119 | 5020d3 | gi-56155383-AY645019.1| | C-5020d3 | Hap_61 |
| 120 | 5022f3 | gi-56155382-AY645018.1| | C-5022f3 | Hap_61 |
| 121 | 5071c3 | gi-56155384-AY645020.1| | C-5071c3 | Hap_61 |
| 122 | 5Ayamcem | D82916 | SE-D5Ay | Hap_59 |
| 123 | 5Baiyiner | AF128324 | 5CD-ba | Hap_21 |
| 124 | 5bankiva | D82899 | 5bankiva | Hap_133 |
| 125 | 5Gallus | AB009436 | 5SE-R | Hap_59 |
| 126 | 5Laihang | AF128325 | 5CD-la | Hap_67 |
| 127 | 5Lingkun | AF128334 | 5CD-L | Hap_21 |
| 128 | 5spadiceu | D82906 | 5SE-spa | Hap_39 |
| 129 | 5varius | D82914 | 5varius | Hap_138 |
| 130 | 5Wugu | AF128339 | 5CD-wu | Hap_21 |
| 131 | 5Xanjuiso | AF128315 | 5CD-x | Hap_3 |
| 132 | 5Xiaoshan | AF128340 | 5CD-xaiso | Hap_61 |
| 133 | 6bankiva | D82897 | 6bankiva | Hap_136 |
| 134 | 6Gallus | AB009435 | 6SE-R | Hap_78 |
| 135 | 6spadeciu | AB007721 | 6SE-spa | Hap_39 |
| 136 | 71Ayam | D82918 | J-71aya | Hap_59 |
| 137 | 7Gallus | AB009434 | 7SE-R | Hap_81 |
| 138 | 83banta | D82922 | Th-83banta | Hap_21 |
| 139 | 8Gallus | AB009433 | 8SE-R | Hap_59 |
| 140 | 9Gallus | AB009432 | 9SE-R | Hap_130 |
| 141 | B1 |  | B1 | Hap_112 |
| 142 | B10 |  | B10 | Hap_112 |
| 143 | B11 |  | B11 | Hap_86 |
| 144 | B12 |  | B12 | Hap_92 |
| 145 | B13 |  | IR-B13 | Hap_122 |
| 146 | B14 |  | IR-B14 | Hap_98 |
| 147 | B15 |  | IR-B15 | Hap_121 |
| 148 | B16 |  | IR-B16 | Hap_92 |
| 149 | B17 |  | IR-B17 | Hap_87 |
| 150 | B18 |  | IR-B18 | Hap_112 |
| 151 | B19 |  | IR-B19 | Hap_111 |
| 152 | B2 |  | IR-B2 | Hap_97 |
| 153 | B20 |  | IR-B20 | Hap_91 |
| 154 | B21 |  | IR-B21 | Hap_97 |
| 155 | B3 |  | IR-B3 | Hap_112 |
| 156 | B4 |  | IR-B4 | Hap_95 |
| 157 | B5 |  | IR-B5 | Hap_112 |
| 158 | B63 |  | IR-B63 | Hap_96 |
| 159 | B7 |  | IR-B7 | Hap_93 |
| 160 | B8 |  | IR-B8 | Hap_89 |
| 161 | B9 |  | IR-B9 | Hap_120 |
| 162 | Barred | D82920 | UK- | Hap_61 |
| 163 | blacksilky.cdhf81 | gi-31296128-AF512067.1| | CD-bsc81 | Hap_50 |
| 164 | blacksilky.cdhf88 | gi-31296129-AF512068.1| | CD-bsc88 | Hap_26 |
| 165 | blacksilky.cdhf89 | gi-31296130-AF512069.1| | CD-bsc89 | Hap_26 |
| 166 | blacksilky.cdhf90 | gi-31296131-AF512070.1| | CD-bsc90 | Hap_3 |
| 167 | blacksilky.cdhf91 | gi-31296132-AF512071.1| | CD-bsc91 | Hap_3 |
| 168 | blacksilky.cdhf93 | gi-31296133-AF512072.1| | CD-bsc93 | Hap_26 |
| 169 | blacksilky.cdhf94 | gi-31296134-AF512073.1| | CD-bsc94 | Hap_10 |
| 170 | blacksilky.cdhf95 | gi-31296135-AF512074.1| | CD-bsc95 | Hap_3 |
| 171 | blacksilky.cdhf96 | gi-31296136-AF512075.1| | CD-bsc96 | Hap_26 |
| 172 | blacksilky.hbhf302 | gi-31296261-AF512200.1| | CD-BS302 | Hap_26 |
| 173 | blacksilky.hbhf304 | gi-31296262-AF512201.1| | CD-BS304 | Hap_16 |
| 174 | blacksilky.hbhf305 | gi-31296263-AF512202.1| | CD-BS305 | Hap_3 |
| 175 | blacksilky.hbhf306 | gi-31296264-AF512203.1| | CD-BS306 | Hap_3 |
| 176 | blacksilky.hbhf307 | gi-31296265-AF512204.1| | CD-BS307 | Hap_26 |
| 177 | blacksilky.hbhf308 | gi-31296266-AF512205.1| | CD-BS308 | Hap_26 |
| 178 | blacksilky.hbhf309 | gi-31296267-AF512206.1| | CD-BS309 | Hap_26 |
| 179 | blacksilky.hbhf310 | gi-31296268-AF512207.1| | CD-BS310 | Hap_26 |
| 180 | blacksilky.hbhf316 | gi-31296269-AF512208.1| | CD-BS316 | Hap_3 |
| 181 | blacksilky.hbhf317 | gi-31296270-AF512209.1| | CD-BS317 | Hap_26 |
| 182 | C1 |  | ID-C1 | Hap_70 |
| 183 | C2 |  | ID-C2 | Hap_68 |
| 184 | C3 |  | ID-C3 | Hap_73 |
| 185 | C4 |  | ID-C4 | Hap_69 |
| 186 | C6 |  | ID-C6 | Hap_71 |
| 187 | Caoke.ck141 | gi-31296152-AF512091.1| | CD-ck141 | Hap_3 |
| 188 | Caoke.ck142 | gi-31296153-AF512092.1| | CD-ck142 | Hap_3 |
| 189 | Caoke.ck143 | gi-31296154-AF512093.1| | CD-ck143 | Hap_3 |
| 190 | Caoke.ck144 | gi-31296155-AF512094.1| | CD-ck144 | Hap_3 |
| 191 | Caoke.ck145 | gi-31296156-AF512095.1| | CD-ck145 | Hap_3 |
| 192 | Caoke.ck146 | gi-31296157-AF512096.1| | CD-ck146 | Hap_3 |
| 193 | Caoke.ck147 | gi-31296158-AF512097.1| | CD-ck147 | Hap_10 |
| 194 | Caoke.ck150 | gi-31296159-AF512098.1| | CD-ck150 | Hap_14 |
| 195 | Caoke.ck153 | gi-31296160-AF512099.1| | CD-ck153 | Hap_3 |
| 196 | Caoke.ck154 | gi-31296161-AF512100.1| | CD-ck154 | Hap_3 |
| 197 | Caoke.ck165 | gi-31296162-AF512101.1| | CD-ck165 | Hap_10 |
| 198 | Caoke.ck167 | gi-31296163-AF512102.1| | CD-ck167 | Hap_3 |
| 199 | Caoke.ck173 | gi-31296164-AF512103.1| | CD-ck173 | Hap_3 |
| 200 | Caoke.cK175 | gi-31296165-AF512104.1| | CD-ck175 | Hap_3 |
| 201 | Caoke.ck177 | gi-31296166-AF512105.1| | CD-ck177 | Hap_3 |
| 202 | Caoke.ck189 | gi-31296167-AF512106.1| | CD-ck189 | Hap_3 |
| 203 | Caoke.ck244 | gi-31296168-AF512107.1| | CD-ck244 | Hap_61 |
| 204 | chabo69 | AB009429 | J-69 | Hap_59 |
| 205 | chaboJap | AB009427 | J-Jap | Hap_3 |
| 206 | Chahua.ch1 | gi-31296137-AF512076.1| | CD-ch1 | Hap_3 |
| 207 | Chahua.ch10 | gi-31296138-AF512077.1| | CD-ch10 | Hap_12 |
| 208 | Chahua.ch11 | gi-31296139-AF512078.1| | CD-ch11 | Hap_12 |
| 209 | Chahua.ch12 | gi-31296140-AF512079.1| | CD-ch12 | Hap_12 |
| 210 | Chahua.ch13 | gi-31296141-AF512080.1| | CD-ch13 | Hap_3 |
| 211 | Chahua.ch14 | gi-31296142-AF512081.1| | CD-ch14 | Hap_3 |
| 212 | Chahua.ch15 | gi-31296143-AF512082.1| | CD-ch15 | Hap_61 |
| 213 | Chahua.ch2 | gi-31296144-AF512083.1| | CD-ch2 | Hap_12 |
| 214 | Chahua.ch3 | gi-31296145-AF512084.1| | CD-ch3 | Hap_26 |
| 215 | Chahua.ch4 | gi-31296146-AF512085.1| | CD-ch4 | Hap_31 |
| 216 | Chahua.ch5 | gi-31296147-AF512086.1| | CD-ch5 | Hap_12 |
| 217 | Chahua.ch6 | gi-31296148-AF512087.1| | CD-ch6 | Hap_26 |
| 218 | Chahua.ch7 | gi-31296149-AF512088.1| | CD-ch7 | Hap_26 |
| 219 | Chahua.ch8 | gi-31296150-AF512089.1| | CD-ch8 | Hap_33 |
| 220 | Chahua.ch9 | gi-31296151-AF512090.1| | CD-ch9 | Hap_12 |
| 221 | CJ | NC_003408 | CJ2 | Hap_47 |
| 222 | D1 | gi-40458093-AY392175.1| | C-D1 | Hap_26 |
| 223 | D12 | gi-40458090-AY392172.1| | C-D12 | Hap_50 |
| 224 | D13 | gi-40458091-AY392173.1| | C-D13 | Hap_37 |
| 225 | D16 | gi-40458092-AY392174.1| | C-D16 | Hap_26 |
| 226 | D20 | gi-40458094-AY392176.1| | C-D20 | Hap_100 |
| 227 | D21 | gi-40458095-AY392177.1| | C-D21 | Hap_26 |
| 228 | D25 | gi-40458096-AY392178.1| | C-D25 | Hap_100 |
| 229 | D26 | gi-40458097-AY392179.1| | C-D26 | Hap_26 |
| 230 | D27 | gi-40458098-AY392180.1| | CD-D27 | Hap_3 |
| 231 | D28 | gi-40458099-AY392181.1| | CD-D28 | Hap_3 |
| 232 | D29 | gi-40458100-AY392182.1| | CD-D29 | Hap_3 |
| 233 | D30 | gi-40458101-AY392183.1| | C-D30 | Hap_26 |
| 234 | D31 | gi-40458102-AY392184.1| | CD-D31 | Hap_3 |
| 235 | D32 | gi-40458103-AY392185.1| | C-D32 | Hap_100 |
| 236 | D33 | gi-40458104-AY392186.1| | CD-D33 | Hap_3 |
| 237 | D34 | gi-40458105-AY392187.1| | C-D34 | Hap_31 |
| 238 | D37 | gi-40458106-AY392188.1| | C-D37 | Hap_26 |
| 239 | D38 | gi-40458107-AY392189.1| | C-D38 | Hap_26 |
| 240 | D39 | gi-40458108-AY392190.1| | CD-D39 | Hap_3 |
| 241 | D40 | gi-40458109-AY392191.1| | C-D40 | Hap_26 |
| 242 | D41 | gi-40458110-AY392192.1| | C-D41 | Hap_37 |
| 243 | D43 | gi-40458111-AY392193.1| | C-D43 | Hap_37 |
| 244 | D46 | gi-40458112-AY392194.1| | C-D46 | Hap_50 |
| 245 | D47 | gi-40458113-AY392195.1| | C-D47 | Hap_26 |
| 246 | D48 | gi-40458114-AY392196.1| | C-D48 | Hap_50 |
| 247 | D49 | gi-40458115-AY392197.1| | C-D49 | Hap_50 |
| 248 | D5 | gi-40458120-AY392202.1| | CD-D5 | Hap_3 |
| 249 | D50 | gi-40458116-AY392198.1| | C-D50 | Hap_26 |
| 250 | D51 | gi-40458117-AY392199.1| | C-D51 | Hap_50 |
| 251 | D52 | gi-40458118-AY392200.1| | C-D52 | Hap_50 |
| 252 | D53 | gi-40458119-AY392201.1| | C-D53 | Hap_50 |
| 253 | D6 | gi-40458121-AY392203.1| | CD-D6 | Hap_3 |
| 254 | D9 | gi-40458122-AY392204.1| | CD-D9 | Hap_3 |
| 255 | Douji.dj1 | gi-31296169-AF512108.1| | CD-d1 | Hap_37 |
| 256 | Douji.dj10 | gi-31296170-AF512109.1| | CD-d10 | Hap_26 |
| 257 | Douji.dj11 | gi-31296171-AF512110.1| | CD-d11 | Hap_26 |
| 258 | Douji.dj12 | gi-31296172-AF512111.1| | CD-d12 | Hap_26 |
| 259 | Douji.dj13 | gi-31296173-AF512112.1| | CD-d13 | Hap_50 |
| 260 | Douji.dj2 | gi-31296174-AF512113.1| | CD-d2 | Hap_3 |
| 261 | Douji.dj5 | gi-31296175-AF512114.1| | CD-d5a | Hap_33 |
| 262 | Douji.dj6 | gi-31296176-AF512115.1| | CD-d6a | Hap_26 |
| 263 | Douji.dj9 | gi-31296177-AF512116.1| | CD-d9a | Hap_26 |
| 264 | Dwarf.dw214 | gi-31296178-AF512117.1| | CD-dw214 | Hap_61 |
| 265 | Dwarf.dw215 | gi-31296179-AF512118.1| | CD-dw215 | Hap_61 |
| 266 | Dwarf.dw237 | gi-31296180-AF512119.1| | CD-dw237 | Hap_3 |
| 267 | Dwarf.dw238 | gi-31296181-AF512120.1| | CD-dw238 | Hap_3 |
| 268 | Dwarf.dw239 | gi-31296182-AF512121.1| | CD-dw239 | Hap_61 |
| 269 | Dwarf.dw240 | gi-31296183-AF512122.1| | CD-dw240 | Hap_3 |
| 270 | Dwarf.dw241 | gi-31296184-AF512123.1| | CD-dw241 | Hap_61 |
| 271 | Dwarf.dw242 | gi-31296185-AF512124.1| | CD-dw242 | Hap_26 |
| 272 | Dwarf.dw243 | gi-31296186-AF512125.1| | CD-dw243 | Hap_26 |
| 273 | Dwarf.dw485 | gi-31296187-AF512126.1| | CD-dw485 | Hap_67 |
| 274 | Dwarf.dw9 | gi-31296188-AF512127.1| | CD-dw9 | Hap_26 |
| 275 | GJ1 |  | GJ1 | Hap_144 |
| 276 | GJ2 |  | GJ2 | Hap_145 |
| 277 | GJ3 |  | GJ3 | Hap_146 |
| 278 | GJ4 |  | GJ4 | Hap_143 |
| 279 | GuizhouMountainWugu.gui178 | gi-31296189-AF512128.1| | CD-GMW1 | Hap_3 |
| 280 | GuizhouMountainWugu.gui181 | gi-31296190-AF512129.1| | CD-GMW10 | Hap_61 |
| 281 | GuizhouMountainWugu.gui182 | gi-31296191-AF512130.1| | CD-GMW11 | Hap_61 |
| 282 | GuizhouMountainWugu.gui183 | gi-31296192-AF512131.1| | CD-GMW2 | Hap_3 |
| 283 | GuizhouMountainWugu.gui184 | gi-31296193-AF512132.1| | CD-GMW3 | Hap_3 |
| 284 | GuizhouMountainWugu.gui185 | gi-31296194-AF512133.1| | CD-GMW4 | Hap_3 |
| 285 | GuizhouMountainWugu.gui186 | gi-31296195-AF512134.1| | CD-GMW5 | Hap_3 |
| 286 | GuizhouMountainWugu.gui187 | gi-31296196-AF512135.1| | CD-GMW6 | Hap_3 |
| 287 | GuizhouMountainWugu.gui188 | gi-31296197-AF512136.1| | CD-GMW7 | Hap_18 |
| 288 | GuizhouMountainWugu.gui190 | gi-31296198-AF512137.1| | CD-GMW8 | Hap_34 |
| 289 | GuizhouMountainWugu.gui191 | gi-31296199-AF512138.1| | CD-GMW9 | Hap_34 |
| 290 | GushiWugu.gushi1 | gi-31296200-AF512139.1| | CD-Gw1 | Hap_61 |
| 291 | GushiWugu.gushi12 | gi-31296201-AF512140.1| | CD-Gw12 | Hap_12 |
| 292 | GushiWugu.gushi13 | gi-31296202-AF512141.1| | CD-Gw13 | Hap_61 |
| 293 | GushiWugu.gushi14 | gi-31296203-AF512142.1| | CD-Gw14 | Hap_61 |
| 294 | GushiWugu.gushi15 | gi-31296204-AF512143.1| | CD-Gw15 | Hap_3 |
| 295 | GushiWugu.gushi16 | gi-31296205-AF512144.1| | CD-Gw16 | Hap_61 |
| 296 | GushiWugu.gushi17 | gi-31296206-AF512145.1| | CD-Gw17 | Hap_3 |
| 297 | GushiWugu.gushi18 | gi-31296207-AF512146.1| | CD-Gw18 | Hap_50 |
| 298 | GushiWugu.gushi19 | gi-31296208-AF512147.1| | CD-Gw19 | Hap_12 |
| 299 | GushiWugu.gushi20 | gi-31296209-AF512148.1| | CD-Gw20 | Hap_61 |
| 300 | GushiWugu.gushi21 | gi-31296210-AF512149.1| | CD-Gw21 | Hap_12 |
| 301 | GushiWugu.gushi26 | gi-31296211-AF512150.1| | CD-Gw26 | Hap_26 |
| 302 | Heikanghk46 | gi-31296271-AF512210.1| | CD46 | Hap_34 |
| 303 | Heikanghk62 | gi-31296272-AF512211.1| | CD62 | Hap_6 |
| 304 | Heikanghk63 | gi-31296273-AF512212.1| | CD63 | Hap_6 |
| 305 | Heikanghk65 | gi-31296274-AF512213.1| | CD65 | Hap_6 |
| 306 | HeikanghK66 | gi-31296275-AF512214.1| | CD66 | Hap_34 |
| 307 | Huxu | gi-31296276-AF512215.1| | CD-h | Hap_34 |
| 308 | Huxuhx380 | gi-31296277-AF512216.1| | CD-h380 | Hap_34 |
| 309 | Huxuhx431 | gi-31296278-AF512217.1| | CD-h431 | Hap_34 |
| 310 | Huxuhx432 | gi-31296279-AF512218.1| | CD-h432 | Hap_34 |
| 311 | Huxuhx433 | gi-31296280-AF512219.1| | CD-h433 | Hap_34 |
| 312 | Huxuhx434 | gi-31296281-AF512220.1| | CD-h434 | Hap_34 |
| 313 | indiaG1 | gi-56155330-AY644966.1| | InD-G1 | Hap_61 |
| 314 | indiaG2 | gi-56155331-AY644967.1| | InD-G2 | Hap_66 |
| 315 | indiaG3 | gi-56155332-AY644968.1| | InD-G3 | Hap_61 |
| 316 | indiaG4 | gi-56155333-AY644969.1| | InD-G4 | Hap_66 |
| 317 | indiaG6 | gi-56155334-AY644970.1| | InD-G6 | Hap_58 |
| 318 | indiaG7 | gi-56155335-AY644971.1| | InD-G7 | Hap_58 |
| 319 | indiaG8 | gi-56155336-AY644972.1| | InD-G8 | Hap_26 |
| 320 | indiaG9 | gi-56155337-AY644973.1| | InD-G9 | Hap_57 |
| 321 | Iran169 | AB009444 | Iran169 | Hap_6 |
| 322 | J1 |  | ID-J1 | Hap_63 |
| 323 | J12 | gi-40458123-AY392205.1| | C-J12 | Hap_37 |
| 324 | J13 | gi-40458124-AY392206.1| | C-J13 | Hap_37 |
| 325 | J14 | gi-40458125-AY392207.1| | C-J14 | Hap_37 |
| 326 | J15 | gi-40458126-AY392208.1| | C-J15 | Hap_37 |
| 327 | J16 | gi-40458127-AY392209.1| | C-J16 | Hap_50 |
| 328 | J17 | gi-40458128-AY392210.1| | C-J17 | Hap_50 |
| 329 | J18 | gi-40458129-AY392211.1| | C-J18 | Hap_37 |
| 330 | J19 | gi-40458130-AY392212.1| | C-J19 | Hap_37 |
| 331 | J2 |  | ID-J2 | Hap_6 |
| 332 | J20 | gi-40458131-AY392213.1| | C-J20 | Hap_42 |
| 333 | J27 | gi-40458132-AY392214.1| | C-J27 | Hap_37 |
| 334 | J29 | gi-40458133-AY392215.1| | C-J29 | Hap_54 |
| 335 | J3 |  | ID-J3 | Hap_63 |
| 336 | J30 | gi-40458134-AY392216.1| | C-J30 | Hap_54 |
| 337 | J31 | gi-40458135-AY392217.1| | CD-J31 | Hap_26 |
| 338 | J32 | gi-40458136-AY392218.1| | C-J32 | Hap_37 |
| 339 | J33 | gi-40458137-AY392219.1| | CD-J33 | Hap_26 |
| 340 | J34 | gi-40458138-AY392220.1| | C-J34 | Hap_56 |
| 341 | J35 | gi-40458139-AY392221.1| | C-J35 | Hap_37 |
| 342 | J36 | gi-40458140-AY392222.1| | C-J36 | Hap_50 |
| 343 | J37 | gi-40458141-AY392223.1| | C-J37 | Hap_31 |
| 344 | J38 | gi-40458142-AY392224.1| | C-J38 | Hap_56 |
| 345 | J39 | gi-40458143-AY392225.1| | C-J39 | Hap_56 |
| 346 | J4 |  | ID-J4 | Hap_63 |
| 347 | J40 | gi-40458144-AY392226.1| | C-J40 | Hap_50 |
| 348 | J41 | gi-40458145-AY392227.1| | C-J41 | Hap_61 |
| 349 | J42 | gi-40458146-AY392228.1| | C-J42 | Hap_37 |
| 350 | J43 | gi-40458147-AY392229.1| | J43 | Hap_25 |
| 351 | J5 |  | J5 | Hap_42 |
| 352 | J5sec | gi-40458148-AY392230.1| | ID-J5sec | Hap_63 |
| 353 | J6 | gi-40458149-AY392231.1| | C-J6 | Hap_50 |
| 354 | J7 | gi-40458150-AY392232.1| | C-J7 | Hap_50 |
| 355 | jabouile1 | gi-31296212-AF512151.1| | C-Ja1 | Hap_19 |
| 356 | jabouile2 | gi-31296213-AF512152.1| | C-Ja2 | Hap_61 |
| 357 | jabouile3 | gi-31296214-AF512153.1| | C-Ja3 | Hap_37 |
| 358 | Jap118ma | AB007742 | J-118ma | Hap_124 |
| 359 | Jap122sh | AB007738 | J-122sh | Hap_61 |
| 360 | Jap128hi | AB007758 | J-128hi | Hap_6 |
| 361 | Jap131na | AB007748 | J-131na | Hap_3 |
| 362 | Jap1696 | AB009445 | J-1696 | Hap_3 |
| 363 | Jap17tok | AB007737 | J-17tok | Hap_76 |
| 364 | Jap192sa | AB007750 | J-192sa | Hap_61 |
| 365 | Jap193sa | AB007747 | J-193sa | Hap_61 |
| 366 | Jap195sa | AB007751 | J-195sa | Hap_3 |
| 367 | Jap196sa | AB007731 | J-196sa | Hap_3 |
| 368 | Jap198sa | AB007732 | J-198sa | Hap_21 |
| 369 | Jap1utha | AB007741 | J-1utha | Hap_127 |
| 370 | Jap201sa | AB007739 | J-201sa | Hap_82 |
| 371 | Jap2nago | AB007730 | J-2nago | Hap_11 |
| 372 | Jap34tho | AB007739 | J-34tho | Hap_34 |
| 373 | Jap37sil | AB007740 | J-37sil | Hap_34 |
| 374 | Jap38sil | AB007733 | J-38sil | Hap_59 |
| 375 | Jap39sil | AB007746 | J-39sil | Hap_34 |
| 376 | Jap40sil | AB007754 | J-40sil | Hap_61 |
| 377 | Jap43sat | AB007755 | J-43sat | Hap_3 |
| 378 | Jap52Pek | AB007749 | J-52Pek | Hap_3 |
| 379 | Jap56syo | AB007745 | J-56syo | Hap_82 |
| 380 | Jap58ona | AB007743 | J-58ona | Hap_34 |
| 381 | Jap71aya | D82918 | SE-D71ay | Hap_129 |
| 382 | Jap81rik | AB007729 | J-81rik | Hap_76 |
| 383 | Jap8thai | AB007724 | J-8thai | Hap_21 |
| 384 | jap98ryu | AB007744 | j-98ryu | Hap_21 |
| 385 | Japan153 | AB009447 | J-j153 | Hap_3 |
| 386 | Japbuff | AB007734 | J-Jbuff | Hap_61 |
| 387 | K1 |  | IR-K1 | Hap_112 |
| 388 | K10 |  | IR-K10 | Hap_112 |
| 389 | K2 |  | IR-K2 | Hap_123 |
| 390 | K3 |  | IR-K3 | Hap_112 |
| 391 | K4 |  | IR-K4 | Hap_112 |
| 392 | K5 |  | IR-K5 | Hap_99 |
| 393 | K6 |  | IR-K6 | Hap_97 |
| 394 | K7 |  | IR-K7 | Hap_112 |
| 395 | K8 |  | IR-K8 | Hap_112 |
| 396 | K9 |  | IR-K9 | Hap_112 |
| 397 | kos10 | AB098670 | J-k10 | Hap_3 |
| 398 | kos13 | AB098671 | J-k13 | Hap_3 |
| 399 | kos14 | AB098672 | J-k14 | Hap_3 |
| 400 | kos15 | AB098673 | J-k15 | Hap_3 |
| 401 | kos3 | AB098674 | J-k3 | Hap_85 |
| 402 | kos53 | AB098675 | J-k53 | Hap_85 |
| 403 | kos77 | AB098676 | J-k77 | Hap_3 |
| 404 | kos78 | AB098687 | J-k78 | Hap_3 |
| 405 | kos79 | AB098677 | J-k79 | Hap_3 |
| 406 | L10 | gi-40458151-AY392233.1| | CD-L10 | Hap_9 |
| 407 | L11 | gi-40458152-AY392234.1| | C-L11 | Hap_50 |
| 408 | L12 | gi-40458153-AY392235.1| | C-L12 | Hap_31 |
| 409 | L13 | gi-40458154-AY392236.1| | C-L13 | Hap_61 |
| 410 | L14 | gi-40458155-AY392237.1| | C-L14 | Hap_50 |
| 411 | L16 | gi-40458156-AY392238.1| | C-L16 | Hap_61 |
| 412 | L18 | gi-40458157-AY392239.1| | C-L18 | Hap_50 |
| 413 | L19 | gi-40458158-AY392240.1| | C-L19 | Hap_31 |
| 414 | L20 | gi-40458159-AY392241.1| | C-L20 | Hap_50 |
| 415 | L22 | gi-40458160-AY392242.1| | C-L22 | Hap_31 |
| 416 | L23 | gi-40458161-AY392243.1| | C-L23 | Hap_31 |
| 417 | L25 | gi-40458162-AY392244.1| | C-L25 | Hap_34 |
| 418 | L26 | gi-40458163-AY392245.1| | C-L26 | Hap_37 |
| 419 | L27 | gi-40458164-AY392246.1| | C-L27 | Hap_50 |
| 420 | L30 | gi-40458165-AY392247.1| | CD-L30 | Hap_3 |
| 421 | L31 | gi-40458166-AY392248.1| | CD-L31 | Hap_5 |
| 422 | L35 | gi-40458167-AY392249.1| | C-L35 | Hap_37 |
| 423 | L4 | gi-40458171-AY392253.1| | C-L4 | Hap_37 |
| 424 | L43 | gi-40458168-AY392250.1| | C-L43 | Hap_37 |
| 425 | L44 | gi-40458169-AY392251.1| | C-L44 | Hap_52 |
| 426 | L45 | gi-40458170-AY392252.1| | C-L45 | Hap_52 |
| 427 | L54 | gi-40458172-AY392254.1| | CD-L54 | Hap_3 |
| 428 | L55 | gi-40458173-AY392255.1| | C-L55 | Hap_50 |
| 429 | L57 | gi-40458174-AY392256.1| | CD-L57 | Hap_3 |
| 430 | L58 | gi-40458175-AY392257.1| | C-L58 | Hap_37 |
| 431 | L6 | gi-40458177-AY392259.1| | CD-L6 | Hap_3 |
| 432 | L60 | gi-40458176-AY392258.1| | CD-L60 | Hap_3 |
| 433 | L8 | gi-40458178-AY392260.1| | C-L8 | Hap_52 |
| 434 | L9 | gi-40458179-AY392261.1| | C-L9 | Hap_61 |
| 435 | Laos696 | AB009448 | Laos696 | Hap_3 |
| 436 | LukeeggWugu_lk1 | gi-31296298-AF512237.1| | CD-LW1 | Hap_18 |
| 437 | LukeeggWugu_lk31 | gi-31296299-AF512238.1| | CD-LW31 | Hap_3 |
| 438 | LukeeggWugu_lk32 | gi-31296300-AF512239.1| | CD-LW32 | Hap_3 |
| 439 | LukeeggWugu_lk34 | gi-31296301-AF512240.1| | CD-LW34 | Hap_3 |
| 440 | LukeeggWugu_lk35 | gi-31296302-AF512241.1| | CD-LW35 | Hap_34 |
| 441 | LukeeggWugu_lk36 | gi-31296303-AF512242.1| | CD-LW36 | Hap_34 |
| 442 | LukeeggWugu_lk37 | gi-31296304-AF512243.1| | CD-LW37 | Hap_26 |
| 443 | LukeeggWugu_lk38 | gi-31296305-AF512244.1| | CD-LW38 | Hap_34 |
| 444 | LukeeggWugu_lk39 | gi-31296306-AF512245.1| | CD-LW39 | Hap_29 |
| 445 | M1 |  | ID-M1 | Hap_77 |
| 446 | M10 |  | ID-M10 | Hap_74 |
| 447 | M15 |  | ID-M15 | Hap_75 |
| 448 | M17 |  | ID-M17 | Hap_74 |
| 449 | M18 |  | ID-M18 | Hap_74 |
| 450 | M8 |  | ID-M8 | Hap_72 |
| 451 | N1 | gi-40458187-AY392269.1| | C-N1 | Hap_61 |
| 452 | N10 | gi-40458180-AY392262.1| | C-N10 | Hap_42 |
| 453 | N11 | gi-40458181-AY392263.1| | C-N11 | Hap_50 |
| 454 | N12 | gi-40458182-AY392264.1| | C-N12 | Hap_42 |
| 455 | N13 | gi-40458183-AY392265.1| | C-N13 | Hap_50 |
| 456 | N14 | gi-40458184-AY392266.1| | C-N14 | Hap_50 |
| 457 | N15 | gi-40458185-AY392267.1| | C-N15 | Hap_42 |
| 458 | N16 | gi-40458186-AY392268.1| | C-N16 | Hap_50 |
| 459 | N2 | gi-40458196-AY392278.1| | C-N2 | Hap_37 |
| 460 | N20 | gi-40458188-AY392270.1| | C-N20 | Hap_50 |
| 461 | N21 | gi-40458189-AY392271.1| | C-N21 | Hap_50 |
| 462 | N24 | gi-40458190-AY392272.1| | C-N24 | Hap_50 |
| 463 | N25 | gi-40458191-AY392273.1| | C-N25 | Hap_42 |
| 464 | N26 | gi-40458192-AY392274.1| | C-N26 | Hap_50 |
| 465 | N27 | gi-40458193-AY392275.1| | C-N27 | Hap_42 |
| 466 | N28 | gi-40458194-AY392276.1| | C-N28 | Hap_50 |
| 467 | N29 | gi-40458195-AY392277.1| | C-N29 | Hap_42 |
| 468 | N3 | gi-40458201-AY392283.1| | C-N3 | Hap_50 |
| 469 | N32 | gi-40458197-AY392279.1| | C-N32 | Hap_50 |
| 470 | N33 | gi-40458198-AY392280.1| | C-N33 | Hap_50 |
| 471 | N37 | gi-40458199-AY392281.1| | C-N37 | Hap_42 |
| 472 | N38 | gi-40458200-AY392282.1| | C-N38 | Hap_49 |
| 473 | N4 | gi-40458206-AY392288.1| | C-N4 | Hap_42 |
| 474 | N42 | gi-40458202-AY392284.1| | C-N42 | Hap_50 |
| 475 | N43 | gi-40458203-AY392285.1| | C-N43 | Hap_50 |
| 476 | N46 | gi-40458204-AY392286.1| | C-N46 | Hap_42 |
| 477 | N47 | gi-40458205-AY392287.1| | C-N47 | Hap_42 |
| 478 | N5 | gi-40458209-AY392291.1| | CD-N5 | Hap_3 |
| 479 | N50 | gi-40458207-AY392289.1| | C-N50 | Hap_50 |
| 480 | N51 | gi-40458208-AY392290.1| | C-N51 | Hap_55 |
| 481 | N6 | gi-40458210-AY392292.1| | C-N6 | Hap_51 |
| 482 | N7 | gi-40458211-AY392293.1| | C-N7 | Hap_50 |
| 483 | N8 | gi-40458212-AY392294.1| | C-N8 | Hap_37 |
| 484 | N9 | gi-40458213-AY392295.1| | C-N9 | Hap_50 |
| 485 | Nixi_nx10 | gi-31296307-AF512246.1| | CD-NX10 | Hap_50 |
| 486 | Nixi_nx11 | gi-31296308-AF512247.1| | CD-NX11 | Hap_10 |
| 487 | Nixi_nx20 | gi-31296309-AF512248.1| | CD-NX20 | Hap_37 |
| 488 | Nixi_nx23 | gi-31296310-AF512249.1| | CD-NX23 | Hap_50 |
| 489 | Nixi_nx3 | gi-31296311-AF512250.1| | CD-NX3 | Hap_33 |
| 490 | Nixi_nx33 | gi-31296312-AF512251.1| | CD-NX33 | Hap_26 |
| 491 | Nixi_nx5 | gi-31296313-AF512252.1| | CD-NX5 | Hap_3 |
| 492 | Nixi_nx7 | gi-31296314-AF512253.1| | CD-NX7 | Hap_3 |
| 493 | Nixi_nx9 | gi-31296315-AF512254.1| | CD-NX9 | Hap_31 |
| 494 | QinyuanMaji_qiny365 | gi-31296316-AF512255.1| | CD-Qm36 | Hap_34 |
| 495 | QinyuanMaji_qiny366 | gi-31296317-AF512256.1| | CD-Qm36a | Hap_34 |
| 496 | QinyuanMaji_qiny367 | gi-31296318-AF512257.1| | CD-Qm36b | Hap_34 |
| 497 | QinyuanMaji_qiny368 | gi-31296319-AF512258.1| | CD-Qm36c | Hap_34 |
| 498 | QinyuanMaji_qiny378 | gi-31296320-AF512259.1| | CD-Qm37 | Hap_34 |
| 499 | QinyuanMaji_qiny379 | gi-31296321-AF512260.1| | CD-Qm37a | Hap_34 |
| 500 | RJ1 |  | IR-RJ1 | Hap_105 |
| 501 | RJ10 |  | IR-RJ10 | Hap_110 |
| 502 | RJ11 |  | IR-RJ11 | Hap_106 |
| 503 | RJ12 |  | IR-RJ12 | Hap_115 |
| 504 | RJ13 |  | IR-RJ13 | Hap_109 |
| 505 | RJ14 |  | IR-RJ14 | Hap_112 |
| 506 | RJ15 |  | IR-RJ15 | Hap_88 |
| 507 | RJ16 |  | IR-RJ16 | Hap_99 |
| 508 | RJ17 |  | IR-RJ17 | Hap_104 |
| 509 | RJ18 |  | IR-RJ18 | Hap_112 |
| 510 | RJ19 |  | IR-RJ19 | Hap_6 |
| 511 | RJ2 |  | IR-RJ2 | Hap_94 |
| 512 | RJ20 |  | IR-RJ20 | Hap_112 |
| 513 | RJ21 |  | IR-RJ21 | Hap_112 |
| 514 | RJ22 |  | IR-RJ22 | Hap_107 |
| 515 | RJ23 |  | IR-RJ23 | Hap_118 |
| 516 | RJ24 |  | IR-RJ24 | Hap_103 |
| 517 | RJ25 |  | IR-RJ25 | Hap_90 |
| 518 | RJ3 |  | IR-RJ3 | Hap_116 |
| 519 | RJ4 |  | IR-RJ4 | Hap_117 |
| 520 | RJ5 |  | IR-RJ5 | Hap_102 |
| 521 | RJ6 |  | IR-RJ6 | Hap_119 |
| 522 | RJ7 |  | IR-RJ7 | Hap_113 |
| 523 | RJ8 |  | IR-RJ8 | Hap_108 |
| 524 | RJ9 |  | IR-RJ9 | Hap_114 |
| 525 | sha100 | AB098641 | J-sh100 | Hap_82 |
| 526 | sha101 | AB098644 | J-sh101 | Hap_82 |
| 527 | sha102 | AB098645 | J-sh102 | Hap_3 |
| 528 | sha111 | AB098651 | J-sh111 | Hap_82 |
| 529 | sha112 | AB098652 | J-sh112 | Hap_21 |
| 530 | sha113 | AB098653 | J-sh113 | Hap_101 |
| 531 | sha114 | AB098697 | J-sh114 | Hap_84 |
| 532 | sha115 | AB098654 | J-sh115 | Hap_101 |
| 533 | sha116 | AB098655 | J-sh116 | Hap_101 |
| 534 | sha117 | AB098698 | J-sh117 | Hap_82 |
| 535 | sha119 | AB098699 | J-sh119 | Hap_84 |
| 536 | sha121 | AB098657 | J-sh121 | Hap_34 |
| 537 | sha122 | AB098658 | J-sh122 | Hap_21 |
| 538 | sha123 | AB098659 | J-sh123 | Hap_101 |
| 539 | sha124 | AB098660 | J-sh124 | Hap_82 |
| 540 | sha125 | AB098661 | J-sh125 | Hap_101 |
| 541 | sha126 | AB098662 | J-sh126 | Hap_101 |
| 542 | sha127 | AB098663 | J-sh127 | Hap_101 |
| 543 | sha148 | AB098683 | J-sh148 | Hap_3 |
| 544 | sha149 | AB098685 | J-sh149 | Hap_21 |
| 545 | sha152 | AB098686 | J-sh152 | Hap_82 |
| 546 | sha170 | AB098682 | J-sh170 | Hap_21 |
| 547 | sha172 | AB098684 | J-sh172 | Hap_21 |
| 548 | sha178 | AB098689 | J-sh178 | Hap_85 |
| 549 | sha180 | AB098690 | J-sh180 | Hap_34 |
| 550 | sha181 | AB098691 | J-sh181 | Hap_3 |
| 551 | sha184 | AB098692 | J-sh184 | Hap_82 |
| 552 | sha185 | AB098693 | J-sh185 | Hap_82 |
| 553 | sha188 | AB098694 | J-sh188 | Hap_82 |
| 554 | sha191 | AB098695 | J-sh191 | Hap_82 |
| 555 | sha24 | AB098636 | J-sh24 | Hap_34 |
| 556 | sha25 | AB098637 | J-sh25 | Hap_83 |
| 557 | sha49 | AB098647 | J-s49 | Hap_82 |
| 558 | sha51 | AB098648 | J-s51 | Hap_34 |
| 559 | sha65 | AB098638 | J-s65 | Hap_101 |
| 560 | sha66 | AB098639 | J-s66 | Hap_101 |
| 561 | sha71 | AB098649 | J-s71 | Hap_82 |
| 562 | sha74 | AB098681 | J-s74 | Hap_82 |
| 563 | sha75 | AB098650 | J-s75 | Hap_3 |
| 564 | sha81 | AB098642 | J-s81 | Hap_34 |
| 565 | sha82 | AB098664 | J-s82 | Hap_23 |
| 566 | sha83 | AB098665 | J-s83 | Hap_23 |
| 567 | sha84 | AB098666 | J-s84 | Hap_3 |
| 568 | sha85 | AB098667 | J-s85 | Hap_24 |
| 569 | sha86 | AB098668 | J-s86 | Hap_23 |
| 570 | sha98 | AB098646 | J-s98 | Hap_101 |
| 571 | sha99 | AB098643 | J-s99 | Hap_3 |
| 572 | shenggoua1849 | gi-31296118-AF512057.1| | CD-g1849 | Hap_38 |
| 573 | shenggoua1850 | gi-31296119-AF512058.1| | CD-g1850 | Hap_38 |
| 574 | shenggoua1851 | gi-31296120-AF512059.1| | CD-s1851 | Hap_3 |
| 575 | SichuanMountainWugu_sdw243 | gi-31296322-AF512261.1| | CD-SMW1 | Hap_3 |
| 576 | SichuanMountainWugu_sdw470 | gi-31296323-AF512262.1| | CD-SMW10 | Hap_61 |
| 577 | SichuanMountainWugu_sdw472 | gi-31296324-AF512263.1| | CD-SMW11 | Hap_61 |
| 578 | SichuanMountainWugu_sdw473 | gi-31296325-AF512264.1| | CD-SMW12 | Hap_67 |
| 579 | SichuanMountainWugu_sdw474 | gi-31296326-AF512265.1| | CD-SMW2 | Hap_3 |
| 580 | SichuanMountainWugu_sdw475 | gi-31296327-AF512266.1| | CD-SMW3 | Hap_3 |
| 581 | SichuanMountainWugu_sdw481 | gi-31296328-AF512267.1| | CD-SMW4 | Hap_26 |
| 582 | SichuanMountainWugu_sdw482 | gi-31296329-AF512268.1| | CD-SMW5 | Hap_35 |
| 583 | SichuanMountainWugu_sdw483 | gi-31296330-AF512269.1| | CD-SMW6 | Hap_35 |
| 584 | SichuanMountainWugu_sdw484 | gi-31296331-AF512270.1| | CD-SMW7 | Hap_35 |
| 585 | SichuanMountainWugu_sdw485 | gi-31296332-AF512271.1| | CD-SMW8 | Hap_61 |
| 586 | SichuanMountainWugu_sdw486 | gi-31296333-AF512272.1| | CD-SMW9 | Hap_61 |
| 587 | silky.cdbf122 | gi-31296250-AF512189.1| | CD-scd122 | Hap_3 |
| 588 | silky.cdbf123 | gi-31296251-AF512190.1| | CD-scd123 | Hap_20 |
| 589 | silky.cdbf126 | gi-31296252-AF512191.1| | CD-scd126 | Hap_3 |
| 590 | silky.cdbf127 | gi-31296253-AF512192.1| | CD-scd127 | Hap_3 |
| 591 | silky.cdbf128 | gi-31296254-AF512193.1| | CD-sc128 | Hap_26 |
| 592 | silky.cdbf129 | gi-31296255-AF512194.1| | CD-scd129 | Hap_3 |
| 593 | silky.cdbf143 | gi-31296256-AF512195.1| | CD-scd143 | Hap_10 |
| 594 | Silky_t545 | gi-31296257-AF512196.1| | CD-ST545 | Hap_26 |
| 595 | Silky_t546 | gi-31296258-AF512197.1| | CD-ST546 | Hap_34 |
| 596 | Silky_t547 | gi-31296259-AF512198.1| | CD-ST547 | Hap_26 |
| 597 | Silky_t548 | gi-31296260-AF512199.1| | CD-ST548 | Hap_34 |
| 598 | Silky_t549 | gi-31296282-AF512221.1| | CD-ST549 | Hap_26 |
| 599 | Silky_t550 | gi-31296283-AF512222.1| | CD-ST550 | Hap_3 |
| 600 | Silky_t551 | gi-31296284-AF512223.1| | CD-ST551 | Hap_3 |
| 601 | Silky_t552 | gi-31296285-AF512224.1| | CD-ST552 | Hap_26 |
| 602 | Silky_t555 | gi-31296286-AF512225.1| | CD-ST555 | Hap_26 |
| 603 | Silky_t558 | gi-31296287-AF512226.1| | CD-ST558 | Hap_3 |
| 604 | Silkyhbbf124 | gi-31296288-AF512227.1| | CD-SH124 | Hap_3 |
| 605 | SilkyhBBF301 | gi-31296289-AF512228.1| | CD-SH301 | Hap_26 |
| 606 | Silkyhbbf5 | gi-31296290-AF512229.1| | CD-SH5 | Hap_26 |
| 607 | Silkyhbbf527 | gi-31296291-AF512230.1| | CD-SH527 | Hap_26 |
| 608 | Silkyhbbf528 | gi-31296292-AF512231.1| | CD-SH528 | Hap_34 |
| 609 | Silkyhbbf530 | gi-31296293-AF512232.1| | CD-SH530 | Hap_26 |
| 610 | Silkyhbbf531 | gi-31296294-AF512233.1| | CD-SH531 | Hap_3 |
| 611 | Silkyhbbf533 | gi-31296295-AF512234.1| | CD-SH533 | Hap_26 |
| 612 | Silkyhbbf534 | gi-31296296-AF512235.1| | CD-SH534 | Hap_34 |
| 613 | Silkyhbbf535 | gi-31296297-AF512236.1| | CD-SH535 | Hap_3 |
| 614 | Silkyhbbf536 | gi-31296334-AF512273.1| | CD-SH536 | Hap_3 |
| 615 | Silkyjsw17 | gi-31296335-AF512274.1| | CD-sjs17 | Hap_26 |
| 616 | Silkyjsw277 | gi-31296336-AF512275.1| | CD-sjs277 | Hap_9 |
| 617 | Silkyjsw440 | gi-31296337-AF512276.1| | CD-sjs440 | Hap_26 |
| 618 | Silkyjsw441 | gi-31296338-AF512277.1| | CD-sj441 | Hap_34 |
| 619 | Silkyjsw442 | gi-31296339-AF512278.1| | CD-sj442 | Hap_34 |
| 620 | Silkyjsw443 | gi-31296340-AF512279.1| | CD-sjs443 | Hap_9 |
| 621 | Silkyjsw444 | gi-31296341-AF512280.1| | CD-sj444 | Hap_34 |
| 622 | Silkyjsw445 | gi-31296342-AF512281.1| | CD-sjs445 | Hap_26 |
| 623 | Silkyjsw446 | gi-31296343-AF512282.1| | CD-sjs446 | Hap_3 |
| 624 | Silkyjsw447 | gi-31296121-AF512060.1| | CD-sjs447 | Hap_61 |
| 625 | Silkyjsw448 | gi-31296122-AF512061.1| | CD-sjs448 | Hap_9 |
| 626 | Silkyjsw450 | gi-31296123-AF512062.1| | CD-sjs450 | Hap_15 |
| 627 | Silkyjsw486 | gi-31296124-AF512063.1| | CD-sj486 | Hap_34 |
| 628 | Silkykd335 | gi-31296125-AF512064.1| | CD-sk335 | Hap_26 |
| 629 | Silkykd336 | gi-31296126-AF512065.1| | CD-sk336 | Hap_26 |
| 630 | Silkykd337 | gi-31296127-AF512066.1| | CD-skd337 | Hap_3 |
| 631 | spadiceush100 | gi-31296231-AF512170.1| | SE-spa00 | Hap_32 |
| 632 | spadiceush101 | gi-31296232-AF512171.1| | SE-spa01 | Hap_58 |
| 633 | spadiceush102 | gi-31296233-AF512172.1| | SE-spa02 | Hap_32 |
| 634 | spadiceush103 | gi-31296234-AF512173.1| | SE-spa03 | Hap_32 |
| 635 | spadiceush104 | gi-31296235-AF512174.1| | SE-spa04 | Hap_31 |
| 636 | spadiceush105 | gi-31296236-AF512175.1| | SE-spa05 | Hap_32 |
| 637 | spadiceush106 | gi-31296237-AF512176.1| | SE-spa06 | Hap_32 |
| 638 | spadiceush107 | gi-31296238-AF512177.1| | SE-spa07 | Hap_58 |
| 639 | spadiceush108 | gi-31296239-AF512178.1| | SE-spa08 | Hap_58 |
| 640 | spadiceush109 | gi-31296240-AF512179.1| | SE-spa09 | Hap_32 |
| 641 | spadiceush3 | gi-31296217-AF512156.1| | SE-spa3 | Hap_10 |
| 642 | spadiceush4 | gi-31296218-AF512157.1| | SE-spa4 | Hap_43 |
| 643 | spadiceush5 | gi-31296219-AF512158.1| | SE-spa5 | Hap_32 |
| 644 | spadiceush87 | gi-31296220-AF512159.1| | SE-spa87 | Hap_32 |
| 645 | spadiceush88 | gi-31296221-AF512160.1| | SE-spa88 | Hap_58 |
| 646 | spadiceush89 | gi-31296222-AF512161.1| | SE-spa89 | Hap_45 |
| 647 | spadiceush90 | gi-31296223-AF512162.1| | SE-spa90 | Hap_32 |
| 648 | spadiceush92 | gi-31296224-AF512163.1| | SE-spa92 | Hap_42 |
| 649 | spadiceush93 | gi-31296225-AF512164.1| | SE-spa93 | Hap_32 |
| 650 | spadiceush95 | gi-31296226-AF512165.1| | SE-spa95 | Hap_58 |
| 651 | spadiceush96 | gi-31296227-AF512166.1| | SE-spa96 | Hap_58 |
| 652 | spadiceush97 | gi-31296228-AF512167.1| | SE-spa97 | Hap_31 |
| 653 | spadiceush98 | gi-31296229-AF512168.1| | SE-spa98 | Hap_58 |
| 654 | spadiceush99 | gi-31296230-AF512169.1| | SE-spa99 | Hap_32 |
| 655 | spadiceushong1 | gi-31296215-AF512154.1| | SE-spa1a | Hap_32 |
| 656 | spadiceushong2 | gi-31296216-AF512155.1| | SE-spa2a | Hap_32 |
| 657 | spadiceussp1 | gi-31296241-AF512180.1| | SE-spa1 | Hap_32 |
| 658 | spadiceussp2 | gi-31296242-AF512181.1| | SE-spa2 | Hap_32 |
| 659 | spadiceussp3 | gi-31296243-AF512182.1| | SE-spa3a | Hap_61 |
| 660 | spadiceussp4 | gi-31296244-AF512183.1| | SE-spa4a | Hap_58 |
| 661 | spadiceussp5 | gi-31296245-AF512184.1| | SE-spa5a | Hap_58 |
| 662 | spadiceussp6 | gi-31296246-AF512185.1| | SE-spa6 | Hap_45 |
| 663 | spadiceussp7 | gi-31296247-AF512186.1| | SE-spa7 | Hap_42 |
| 664 | spadiceussp8 | gi-31296248-AF512187.1| | SE-spa9 | Hap_32 |
| 665 | spadiceussp9 | gi-31296249-AF512188.1| | SE-spa8 | Hap_58 |
| 666 | T1 | gi-40458222-AY392304.1| | C-T1 | Hap_31 |
| 667 | T10 | gi-40458214-AY392296.1| | C-T10 | Hap_51 |
| 668 | T12 | gi-40458215-AY392297.1| | C-T12 | Hap_42 |
| 669 | T13 | gi-40458216-AY392298.1| | C-T19 | Hap_51 |
| 670 | T15 | gi-40458217-AY392299.1| | C-T2 | Hap_31 |
| 671 | T16 | gi-40458218-AY392300.1| | C-T20 | Hap_42 |
| 672 | T17 | gi-40458219-AY392301.1| | C-T21 | Hap_31 |
| 673 | T18 | gi-40458220-AY392302.1| | C-T24 | Hap_46 |
| 674 | T19 | gi-40458221-AY392303.1| | C-T25 | Hap_61 |
| 675 | T2 | gi-40458233-AY392315.1| | C-T26 | Hap_42 |
| 676 | T20 | gi-40458223-AY392305.1| | C-T27 | Hap_39 |
| 677 | T21 | gi-40458224-AY392306.1| | C-T28 | Hap_42 |
| 678 | T22 | gi-40458225-AY392307.1| | C-T29 | Hap_42 |
| 679 | T23 | gi-40458226-AY392308.1| | C-T30 | Hap_42 |
| 680 | T24 | gi-40458227-AY392309.1| | C-T32 | Hap_37 |
| 681 | T25 | gi-40458228-AY392310.1| | C-T33 | Hap_39 |
| 682 | T26 | gi-40458229-AY392311.1| | C-T34 | Hap_37 |
| 683 | T27 | gi-40458230-AY392312.1| | C-T36 | Hap_37 |
| 684 | T28 | gi-40458231-AY392313.1| | C-T38 | Hap_39 |
| 685 | T29 | gi-40458232-AY392314.1| | C-T39 | Hap_37 |
| 686 | T30 | gi-40458234-AY392316.1| | C-T45 | Hap_39 |
| 687 | T32 | gi-40458235-AY392317.1| | C-T46 | Hap_41 |
| 688 | T33 | gi-40458236-AY392318.1| | C-T47 | Hap_39 |
| 689 | T34 | gi-40458237-AY392319.1| | C-T48 | Hap_39 |
| 690 | T36 | gi-40458238-AY392320.1| | C-T49 | Hap_42 |
| 691 | T38 | gi-40458239-AY392321.1| | C-T54 | Hap_46 |
| 692 | T39 | gi-40458240-AY392322.1| | C-T55 | Hap_48 |
| 693 | T41 | gi-40458241-AY392323.1| | C-T57 | Hap_61 |
| 694 | T42 | gi-40458242-AY392324.1| | C-T58 | Hap_42 |
| 695 | T43 | gi-40458243-AY392325.1| | C-T59 | Hap_39 |
| 696 | T44 | gi-40458244-AY392326.1| | C-T7 | Hap_42 |
| 697 | T45 | gi-40458245-AY392327.1| | CD-T13 | Hap_3 |
| 698 | T46 | gi-40458246-AY392328.1| | CD-T15 | Hap_3 |
| 699 | T47 | gi-40458247-AY392329.1| | CD-T16 | Hap_3 |
| 700 | T48 | gi-40458248-AY392330.1| | CD-T17 | Hap_8 |
| 701 | T49 | gi-40458249-AY392331.1| | CD-T18 | Hap_3 |
| 702 | T52 | gi-40458250-AY392332.1| | CD-T22 | Hap_3 |
| 703 | T53 | gi-40458251-AY392333.1| | CD-T23 | Hap_3 |
| 704 | T54 | gi-40458252-AY392334.1| | CD-T41 | Hap_3 |
| 705 | T55 | gi-40458253-AY392335.1| | CD-T42 | Hap_3 |
| 706 | T57 | gi-40458254-AY392336.1| | CD-T43 | Hap_3 |
| 707 | T58 | gi-40458255-AY392337.1| | CD-T44 | Hap_3 |
| 708 | T59 | gi-40458256-AY392338.1| | CD-T52 | Hap_3 |
| 709 | T7 | gi-40458257-AY392339.1| | CD-T53 | Hap_3 |
| 710 | T8 | gi-40458258-AY392340.1| | CD-T8 | Hap_3 |
| 711 | vietnam | AB009449 | SE-Dviet | Hap_80 |
| 712 | W1 | gi-40458268-AY392350.1| | CD-W1 | Hap_3 |
| 713 | W10 | gi-40458259-AY392341.1| | CD-W10 | Hap_3 |
| 714 | W11 | gi-40458260-AY392342.1| | C-W11 | Hap_50 |
| 715 | W12 | gi-40458261-AY392343.1| | CD-W12 | Hap_3 |
| 716 | W13 | gi-40458262-AY392344.1| | C-W13 | Hap_34 |
| 717 | W14 | gi-40458263-AY392345.1| | C-W14 | Hap_37 |
| 718 | W16 | gi-40458264-AY392346.1| | C-W16 | Hap_50 |
| 719 | W17 | gi-40458265-AY392347.1| | CD-W17 | Hap_3 |
| 720 | W18 | gi-40458266-AY392348.1| | C-W18 | Hap_50 |
| 721 | W19 | gi-40458267-AY392349.1| | C-W19 | Hap_26 |
| 722 | W2 | gi-40458274-AY392356.1| | CD-W2 | Hap_3 |
| 723 | W21 | gi-40458269-AY392351.1| | C-W21 | Hap_52 |
| 724 | W25 | gi-40458270-AY392352.1| | C-W25 | Hap_50 |
| 725 | W27 | gi-40458271-AY392353.1| | C-W27 | Hap_50 |
| 726 | W28 | gi-40458272-AY392354.1| | C-W28 | Hap_44 |
| 727 | W29 | gi-40458273-AY392355.1| | C-W29 | Hap_50 |
| 728 | W3 | gi-40458282-AY392364.1| | CD-W3 | Hap_3 |
| 729 | W30 | gi-40458275-AY392357.1| | C-W30 | Hap_50 |
| 730 | W31 | gi-40458276-AY392358.1| | C-W31 | Hap_26 |
| 731 | W32 | gi-40458277-AY392359.1| | C-W32 | Hap_37 |
| 732 | W35 | gi-40458278-AY392360.1| | C-W35 | Hap_50 |
| 733 | W36 | gi-40458279-AY392361.1| | C-W36 | Hap_27 |
| 734 | W38 | gi-40458280-AY392362.1| | C-W38 | Hap_27 |
| 735 | W39 | gi-40458281-AY392363.1| | CD-W39 | Hap_3 |
| 736 | W4 | gi-40458286-AY392368.1| | CD-W4 | Hap_3 |
| 737 | W40 | gi-40458283-AY392365.1| | C-W40 | Hap_26 |
| 738 | W41 | gi-40458284-AY392366.1| | C-W41 | Hap_26 |
| 739 | W49 | gi-40458285-AY392367.1| | C-W49 | Hap_26 |
| 740 | W5 | gi-40458290-AY392372.1| | CD-W5 | Hap_3 |
| 741 | W52 | gi-40458287-AY392369.1| | C-W52 | Hap_37 |
| 742 | W53 | gi-40458288-AY392370.1| | C-W53 | Hap_37 |
| 743 | W56 | gi-40458289-AY392371.1| | C-W56 | Hap_30 |
| 744 | W6 | gi-40458291-AY392373.1| | C-W6 | Hap_26 |
| 745 | W7 | gi-40458292-AY392374.1| | CD-W7 | Hap_7 |
| 746 | W8 | gi-40458293-AY392375.1| | CD-W8 | Hap_3 |
| 747 | W9 | gi-40458294-AY392376.1| | C-W9 | Hap_26 |
| 748 | WangfengWugu_wang436 | gi-31296344-AF512283.1| | CD-Ww1 | Hap_10 |
| 749 | WangfengWugu_wangf388 | gi-31296345-AF512284.1| | CD-Ww2 | Hap_10 |
| 750 | WangfengWugu_wangf389 | gi-31296346-AF512285.1| | CD-Ww3 | Hap_10 |
| 751 | WangfengWugu_wangf390 | gi-31296347-AF512286.1| | CD-WW4 | Hap_3 |
| 752 | WangfengWugu_wangf391 | gi-31296348-AF512287.1| | CD-WW6 | Hap_26 |
| 753 | WangfengWugu_wangf392 | gi-31296349-AF512288.1| | CD-WW7 | Hap_26 |
| 754 | WangfengWugu_wangf437 | gi-31296350-AF512289.1| | CD-WW8 | Hap_26 |
| 755 | WangfengWugu_wangf438 | gi-31296351-AF512290.1| | CD-WW9 | Hap_3 |
| 756 | WangfengWugu_wangf439 | gi-31296352-AF512291.1| | CD-WW1a | Hap_50 |
| 757 | WudingWugu_wd1 | gi-31296353-AF512292.1| | CD-WW2a | Hap_33 |
| 758 | WudingWugu_wd2 | gi-31296354-AF512293.1| | CD-Ww4a | Hap_10 |
| 759 | WudingWugu_wd4 | gi-31296355-AF512294.1| | 1CD-Ww | Hap_26 |
| 760 | WudingWugu_wd6 | gi-31296356-AF512295.1| | 3CD-Ww | Hap_34 |
| 761 | WudingWugu_wd7 | gi-31296357-AF512296.1| | 4CD-Ww | Hap_37 |
| 762 | WudingWugu_wd8 | gi-31296358-AF512297.1| | 5CD-Ww | Hap_50 |
| 763 | WudingWugu_wd9 | gi-31296359-AF512298.1| | 6CD-Ww | Hap_50 |
| 764 | X1 | gi-40458303-AY392385.1| | C-X1 | Hap_26 |
| 765 | X10 | gi-40458295-AY392377.1| | C-X10 | Hap_31 |
| 766 | X11 | gi-40458296-AY392378.1| | C-X11 | Hap_26 |
| 767 | X14 | gi-40458297-AY392379.1| | C-X14 | Hap_13 |
| 768 | X15 | gi-40458298-AY392380.1| | C-X15 | Hap_13 |
| 769 | X16 | gi-40458299-AY392381.1| | C-X16 | Hap_13 |
| 770 | X17 | gi-40458300-AY392382.1| | C-X17 | Hap_31 |
| 771 | X18 | gi-40458301-AY392383.1| | C-X18 | Hap_31 |
| 772 | X19 | gi-40458302-AY392384.1| | C-X19 | Hap_31 |
| 773 | X2 | gi-40458306-AY392388.1| | C-X2 | Hap_13 |
| 774 | X21 | gi-40458304-AY392386.1| | C-X21 | Hap_26 |
| 775 | X28 | gi-40458305-AY392387.1| | C-X28 | Hap_26 |
| 776 | X3 | gi-40458312-AY392394.1| | C-X3 | Hap_13 |
| 777 | X30 | gi-40458307-AY392389.1| | C-X30 | Hap_26 |
| 778 | X32 | gi-40458308-AY392390.1| | C-X32 | Hap_26 |
| 779 | X33 | gi-40458309-AY392391.1| | C-X33 | Hap_26 |
| 780 | X35 | gi-40458310-AY392392.1| | C-X35 | Hap_26 |
| 781 | X36 | gi-40458311-AY392393.1| | C-X36 | Hap_31 |
| 782 | X4 | gi-40458313-AY392395.1| | C-X4 | Hap_13 |
| 783 | X5 | gi-40458315-AY392397.1| | C-X5 | Hap_31 |
| 784 | X59 | gi-40458314-AY392396.1| | C-X59 | Hap_13 |
| 785 | X61 | gi-40458316-AY392398.1| | C-X61 | Hap_31 |
| 786 | X62 | gi-40458317-AY392399.1| | C-X62 | Hap_31 |
| 787 | X64 | gi-40458318-AY392400.1| | C-X64 | Hap_13 |
| 788 | X65 | gi-40458319-AY392401.1| | C-X65 | Hap_26 |
| 789 | X66 | gi-40458320-AY392402.1| | C-X66 | Hap_26 |
| 790 | X67 | gi-40458321-AY392403.1| | C-X67 | Hap_31 |
| 791 | X68 | gi-40458322-AY392404.1| | C-X68 | Hap_13 |
| 792 | X69 | gi-40458323-AY392405.1| | C-X69 | Hap_31 |
| 793 | X7 | gi-40458325-AY392407.1| | C-X7 | Hap_31 |
| 794 | X77 | gi-40458324-AY392406.1| | C-X77 | Hap_26 |
| 795 | XuefengWugu_xf1 | gi-31296360-AF512299.1| | CD-xf1 | Hap_30 |
| 796 | XuefengWugu_xf10 | gi-31296361-AF512300.1| | CD-xf10 | Hap_26 |
| 797 | XuefengWugu_xf11 | gi-31296362-AF512301.1| | CD-xf11 | Hap_61 |
| 798 | XuefengWugu_xf14 | gi-31296363-AF512302.1| | CD-xf14 | Hap_34 |
| 799 | XuefengWugu_xf16 | gi-31296364-AF512303.1| | CD-xf16 | Hap_26 |
| 800 | XuefengWugu_xf17 | gi-31296365-AF512304.1| | CD-xf17 | Hap_26 |
| 801 | XuefengWugu_xf7 | gi-31296366-AF512305.1| | CD-xf7 | Hap_26 |
| 802 | XuefengWugu_xf9 | gi-31296367-AF512306.1| | CD-xf9 | Hap_26 |
| 803 | Yaannon-Wugu_yh457 | gi-31296378-AF512317.1| | CD-YW457 | Hap_3 |
| 804 | Yaannon-Wugu_yh458 | gi-31296379-AF512318.1| | CD-YW458 | Hap_3 |
| 805 | Yaannon-Wugu_yh459 | gi-31296380-AF512319.1| | CD-YW459 | Hap_17 |
| 806 | Yaannon-Wugu_yh460 | gi-31296381-AF512320.1| | CD-YW460 | Hap_3 |
| 807 | Yaannon-Wugu_yh461 | gi-31296382-AF512321.1| | CD-YW461 | Hap_6 |
| 808 | Yaannon-Wugu_yh462 | gi-31296383-AF512322.1| | CD-YW462 | Hap_17 |
| 809 | Yaannon-Wugu_yh470 | gi-31296384-AF512323.1| | CD-YW470 | Hap_3 |
| 810 | YaanWugu_yw580 | gi-31296389-AF512328.1| | CD-YW580 | Hap_3 |
| 811 | YaanWugu_yw581 | gi-31296390-AF512329.1| | CD-YW581 | Hap_36 |
| 812 | YaanWugu_yw582 | gi-31296391-AF512330.1| | CD-YW582 | Hap_61 |
| 813 | YaanWugu_yw583 | gi-31296392-AF512331.1| | CD-YW583 | Hap_3 |
| 814 | YaanWugu_yw584 | gi-31296393-AF512332.1| | CD-YW584 | Hap_17 |
| 815 | YaanWugu_yw585 | gi-31296394-AF512333.1| | CD-YW585 | Hap_61 |
| 816 | YaanWugu_yw586 | gi-31296395-AF512334.1| | CD-YW586 | Hap_26 |
| 817 | YaanWugu_yw587 | gi-31296396-AF512335.1| | CD-YW587 | Hap_17 |
| 818 | YaanWugu_yw588 | gi-31296397-AF512336.1| | CD-YW588 | Hap_26 |
| 819 | YaanWugu_yw589 | gi-31296398-AF512337.1| | CD-YW589 | Hap_61 |
| 820 | yam106 | AB098678 | J-y106 | Hap_85 |
| 821 | yam108 | AB098679 | J-y108 | Hap_85 |
| 822 | yam110 | AB098680 | J-y110 | Hap_85 |
| 823 | YanjingWugu_yj1 | gi-31296385-AF512324.1| | CD-yw1 | Hap_34 |
| 824 | YanjingWugu_yJ2 | gi-31296386-AF512325.1| | CD-yw2 | Hap_28 |
| 825 | YanjingWugu_yj3 | gi-31296387-AF512326.1| | CD-Yw3 | Hap_26 |
| 826 | YanjingWugu_yj4 | gi-31296388-AF512327.1| | CD-Yw4 | Hap_50 |
| 827 | YunxianWugu_y1 | gi-31296368-AF512307.1| | CD-Yw1a | Hap_53 |
| 828 | YunxianWugu_y10 | gi-31296369-AF512308.1| | CD-yw10 | Hap_26 |
| 829 | YunxianWugu_y2 | gi-31296370-AF512309.1| | CD-Yw2a | Hap_50 |
| 830 | YunxianWugu_y3 | gi-31296371-AF512310.1| | CD-yw3a | Hap_34 |
| 831 | YunxianWugu_y328 | gi-31296372-AF512311.1| | CD-yw328 | Hap_35 |
| 832 | YunxianWugu_y329 | gi-31296373-AF512312.1| | CD-yw329 | Hap_26 |
| 833 | YunxianWugu_y335 | gi-31296374-AF512313.1| | CD-yw335 | Hap_35 |
| 834 | YunxianWugu_y336 | gi-31296375-AF512314.1| | CD-yw336 | Hap_10 |
| 835 | YunxianWugu_y337 | gi-31296376-AF512315.1| | CD-yw337 | Hap_10 |
| 836 | YunxianWugu_y338 | gi-31296377-AF512316.1| | CD-yw338 | Hap_34 |
| 837 | ID-i-1 | >gi|56684160|gb|AY704701.1 | InD-1 | Hap_61 |
| 838 | ID-i-10 | >gi|56684161|gb|AY704702.1 | InD-10 | Hap_61 |
| 839 | ID-i-11 | >gi|56684162|gb|AY704703.1 | InD-11 | Hap_61 |
| 840 | ID-i-12 | >gi|56684163|gb|AY704704.1 | InD-12 | Hap_61 |
| 841 | ID-i-13 | >gi|56684164|gb|AY704705.1 | InD-13 | Hap_61 |
| 842 | ID-i-14 | >gi|56684165|gb|AY704706.1 | InD-14 | Hap_61 |
| 843 | ID-i-15 | >gi|56684166|gb|AY704707.1 | InD-15 | Hap_61 |
| 844 | ID-i-18 | >gi|56684167|gb|AY704708.1 | InD-18 | Hap_63 |
| 845 | ID-i-19 | >gi|56684168|gb|AY704709.1 | InD-19 | Hap_60 |
| 846 | ID-i-2 | >gi|56684169|gb|AY704710.1 | InD-2 | Hap_61 |
| 847 | ID-i-20 | >gi|56684170|gb|AY704711.1 | InD-20 | Hap_61 |
| 848 | ID-i-21 | >gi|56684171|gb|AY704712.1 | InD-21 | Hap_62 |
| 849 | ID-i-23 | >gi|56684172|gb|AY704713.1 | InD-23 | Hap_61 |
| 850 | ID-i-25 | >gi|56684173|gb|AY704714.1 | InD-25 | Hap_61 |
| 851 | ID-i-3 | >gi|56684174|gb|AY704715.1 | InD-3 | Hap_61 |
| 852 | ID-i-5 | >gi|56684175|gb|AY704716.1 | InD-5 | Hap_61 |
| 853 | ID-i-6 | >gi|56684176|gb|AY704717.1 | InD-6 | Hap_61 |
| 854 | ID-i-8 | >gi|56684177|gb|AY704718.1 | InD-8 | Hap_58 |
| 855 | ID-i-9 | >gi|56684178|gb|AY704719.1 | InD-9 | Hap_59 |
