# Supplementary material for: Genetic evidence from Indian red jungle fowl corroborates multiple domestication of modern day chicken
Source: BMC Evol Biol. 2008 Jun 10;8:174. doi: 10.1186/1471-2148-8-174 (PMC2474866; doi:10.1186/1471-2148-8-174)

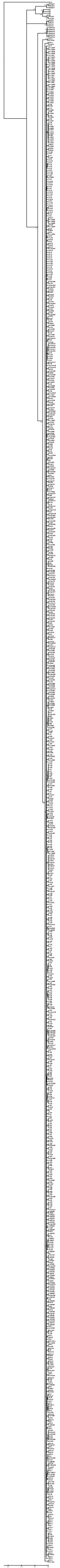

Supplement: Supplementary file 4 [file 1471-2148-8-174-S2.doc]
